# Petrological and experimental evidence for differentiation of water-rich magmas beneath St. Kitts, Lesser Antilles

**DOI:** 10.1007/s00410-017-1416-3

**Published:** 2017-11-10

**Authors:** Elena Melekhova, Jon Blundy, Rita Martin, Richard Arculus, Michel Pichavant

**Affiliations:** 1 School of Earth Sciences, University of Bristol, Wills Memorial Building, Bristol, BS8 1RJ UK; 2 Research School of Earth Sciences, Australian National University, Canberra, ACT 2601 Australia; 3 CNRS/INSU, ISTO, BRGM, UMR 7327, Université d’Orléans, 1A Rue de la Ferollerie, 45071 Orléans, France

**Keywords:** Xenolith, Cumulates, High-An plagioclase, Differentiation of basaltic andesite, Experiments, ‘Magma mush’

## Abstract

St. Kitts lies in the northern Lesser Antilles, a subduction-related intraoceanic volcanic arc known for its magmatic diversity and unusually abundant cognate xenoliths. We combine the geochemistry of xenoliths, melt inclusions and lavas with high pressure–temperature experiments to explore magma differentiation processes beneath St. Kitts. Lavas range from basalt to rhyolite, with predominant andesites and basaltic andesites. Xenoliths, dominated by calcic plagioclase and amphibole, typically in reaction relationship with pyroxenes and olivine, can be divided into plutonic and cumulate varieties based on mineral textures and compositions. Cumulate varieties, formed primarily by the accumulation of liquidus phases, comprise ensembles that represent *instantaneous solid compositions* from one or more magma batches; plutonic varieties have mineralogy and textures consistent with protracted solidification of magmatic mush. Mineral chemistry in lavas and xenoliths is subtly different. For example, plagioclase with unusually high anorthite content (An_≤100_) occurs in some plutonic xenoliths, whereas the most calcic plagioclase in cumulate xenoliths and lavas are An_97_ and An_95_, respectively. Fluid-saturated, equilibrium crystallisation experiments were performed on a St. Kitts basaltic andesite, with three different fluid compositions (*X*H_2_O = 1.0, 0.66 and 0.33) at 2.4 kbar, 950–1025 °C, and *f*O_2_ = NNO − 0.6 to NNO + 1.2 log units. Experiments reproduce lava liquid lines of descent and many xenolith assemblages, but fail to match xenolith and lava phenocryst mineral compositions, notably the very An-rich plagioclase. The strong positive correlation between experimentally determined plagioclase-melt Kd_Ca–Na_ and dissolved H_2_O in the melt, together with the occurrence of Al-rich mafic lavas, suggests that parental magmas were water-rich (> 9 wt% H_2_O) basaltic andesites that crystallised over a wide pressure range (1.5–6 kbar). Comparison of experimental and natural (lava, xenolith) mafic mineral composition reveals that whereas olivine in lavas is predominantly primocrysts precipitated at low-pressure, pyroxenes and spinel are predominantly xenocrysts formed by disaggregation of plutonic mushes. Overall, St. Kitts xenoliths and lavas testify to mid-crustal differentiation of low-MgO basalt and basaltic andesite magmas within a trans-crustal, magmatic mush system. Lower crustal ultramafic cumulates that relate parental low-MgO basalts to primary, mantle -derived melts are absent on St. Kitts.

**Electronic supplementary material:**

The online version of this article (10.1007/s00410-017-1416-3) contains supplementary material, which is available to authorized users.

## Introduction

Arc magmatism above subduction zones involves chemical differentiation of mantle-derived basaltic magmas to intermediate and silicic compositions. Differentiation involves a combination of fractional crystallisation, and crustal melting and assimilation, the relative importance of which varies within and between arcs. Erupted arc magmas represent the integration of differentiation processes that begin with melting in the mantle wedge and, consequently, provide information only on an end product. A complementary approach is to focus on xenoliths brought to the surface during eruptions (e.g. Baker 1968; Arculus and Wills 1980; Conrad et al. 1983; Kay and Kay 1985; Hickey-Vargas et al. 1995; Ducea and Saleeby 1998; Costa et al. 2002; Dungan and Davidson 2004; Mcleod et al. 2013; Yamamoto et al. 2013; Smith 2014; Haase et al. 2014; Price et al. 2016). Such xenoliths may include cumulate residues from crystal fractionation, plutonic equivalents of erupted magma, or fragments of crustal rocks. Here we focus on xenoliths found on the Lesser Antilles island of St. Kitts. By integrating insights from xenoliths with the geochemical record of volcanic rocks and with high pressure–temperature phase equilibrium experiments, we develop an image of the sub-volcanic magma plumbing system beneath St. Kitts.

### Geological setting and previous work

St. Kitts lies within the central Lesser Antilles volcanic arc formed by westwards subduction of the Atlantic oceanic lithosphere. The geology of the island is described in some detail by Baker (1968, 1984) and Toothill et al. (2007). In brief, St. Kitts comprises four volcanic centers, the most prominent of which is Mt. Liamuiga (formerly Mt. Misery) rising 1157 m above sea-level. The oldest volcanic rocks of the island have been dated at ~ 1–2 Ma (Maury and Westercamp 1990), although radiometric ages are rather sparse. The last dated eruption, from Mt. Liamuiga, was 1800 years BP and there have been no documented eruptions since settlement in 1624. Erupted rock types range from basalt to rhyolite, with basaltic andesites and andesites dominant (Baker 1984), both as pyroclastic deposits and as lavas. Magnesium-rich basalt (≤ 7 wt% MgO) lavas, with phenocrysts of olivine, clinopyroxene, and plagioclase, occur on the north-east coast at Black Rocks. These are putative parental magmas (Turner et al. 1996; Toothill et al. 2007), although their Mg# (molar Mg/[Mg + Fe]) ≤ 0.64 are too low to have been derived directly from mantle wedge peridotite (Toothill et al. 2007). Isotopic data indicate that differentiation was dominated by fractional crystallisation processes with negligible assimilation of older sialic crust and limited (< 10%) assimilation of biogenic sediments (Toothill et al. 2007). Consequently, St. Kitts represents one extreme of arc magmatic differentiation in which the role of crustal melting is minimal.

In keeping with other Lesser Antilles volcanic islands (Arculus and Wills 1980), St. Kitts yields a large number of magmatic (or cognate) xenoliths, entrained in pyroclastic rocks. Xenoliths from St. Kitts were first described by Fels (1903) and Earle (1925). Baker (1968) notes that St. Kitts xenoliths occur originally in pyroclastic rocks, but are also preferentially weathered out and accumulate in river drainage channels (or “ghuts”). Although such samples lack geological context, in terms of their parent eruptions, their accumulation provides a means to sample a great variety of textural and chemical types. Xenoliths, with or without fragments of host lava, occur as rounded clasts ranging in size from few cm to a half a metre.

Given the relative youth of the island, all xenoliths can be ascribed to the magmatic activity that constructed St. Kitts and are, therefore, representative of the sub-volcanic arc crust. Baker (1968) presents petrographic data for thirteen xenoliths from Harris, Godwin, Saddler’s and Pogson’s Ghuts. A further thirteen xenoliths were described in detail by Arculus and Wills (1980). St. Kitts xenoliths exhibit great petrological diversity, with assemblages including: (1) olivine + plagioclase; (2) olivine + plagioclase + orthopyroxene + magnetite; (3) olivine + plagioclase + orthopyroxene + clinopyroxene + magnetite + amphibole; and (4) plagioclase + orthopyroxene + clinopyroxene + magnetite + amphibole + quartz + biotite. The combination of relatively abundant orthopyroxene and very calcic plagioclase (> 94 mol% anorthite) distinguishes St. Kitts xenoliths from other Lesser Antilles islands (Baker 1968; Lewis 1973; Arculus and Wills 1980; Kiddle et al. 2010; Tollan et al. 2012; Stamper et al. 2014; Cooper et al. 2017).

This study is based on a total of 35 St. Kitts xenoliths collected during a field campaign in 2009 and augmented by five xenoliths from the collection at Durham University. Petrological data from St. Kitts xenoliths are studied in conjunction with published whole-rock geochemical analyses of St. Kitts lavas (Baker 1984; Turner et al. 1996; Toothill et al. 2007) and new and published experimental petrology data.

## Methods

### Analytical

**Fig. 1:**
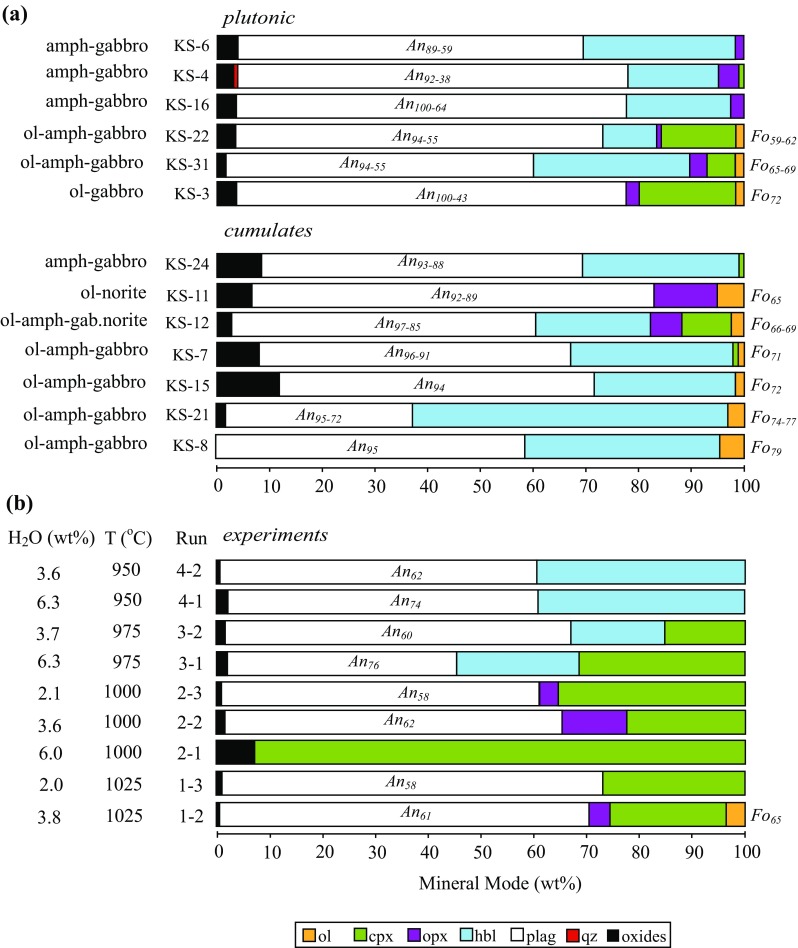
**a** Modal proportions, by mass, of minerals in St. Kitts cumulate xenoliths using the classification in Table 1. Cumulates are listed (from bottom to the top) in order of decreasing Fo content of olivine, followed by An of plagioclase. **b** Modal proportions of silicate and oxide minerals in experimental solid residues

Initial petrographic analyses were carried out on forty St. Kitts xenoliths. These were subsequently divided into thirteen representative types based on mineral assemblages and textures (Table 1). Xenoliths were classified using the British Geological Society Rock Classification Scheme (1999), and Streckeisen (1976). Modal abundances of the major mineral phases for each of the 13 representative xenoliths were obtained by point counting (Table 1, Fig. 1) using a Pelcon Automatic point counter 1.8 coupled to an optical microscope. Between 1250 and 1750 points were counted for each xenolith. Volume modes were converted into mass modes using mineral densities, modified where appropriate for solid solution (as determined from mineral analyses), from Deer et al. (1992).

**Table 1:** Classification, modes, and brief descriptions of xenolith samples

Sample	Rock type	Mineral modes (wt %)	Description
*Cumulates*			
KS-8	ol-amph-gabbro	ol (5), amph (37), pl (58)	Coarse-grained; euhedral, unzoned, incl- and MI-rich pl ≤ 20 mm; enclosed by subhedral amph; interstitial scoria
KS-21	ol-amph-gabbro	ol (3) amph (60), pl (35), mag (2), sulf (tr)	Very coarse-grained; large poikilitic amph; unzoned euhedral pl with MIs
KS-15	ol-amph-gabbro	ol (2), amph (27), pl (60), mag (12), sulf (tr)	Coarse-grained; mag ≤ 20 mm, some with sulf inclusions; amph and pl ≤ 30 mm; iddingsitised ol; amph and pl rich in MIs
KS-7	ol-amph-gabbro	ol (1), cpx (1), amph (31), pl (59), mag (8), sulf (tr)	Coarse-grained; large poikilitic amph; incl-rich but unzoned pl; ol surrounded by amph; cpx with Mis; sulf in mt and as separate grains, interstitial scoria
KS-12	ol-amph-gabbronorite	ol (2), cpx (9), opx (6), amph (21), pl (58), mag (3), sulf (tr), bio (tr)	Fine-to-coarse-grained; Two generations of amph some poikilitic enclosing cpx and ol, others euhedral enclosed by pl; MIs in opx
KS-11	ol-norite	ol (5), opx (12), pl (77), mag (6), sulf (tr)	Coarse-grained; equilibrated (120o intersections); unzoned MI-bearing pl and opx
KS-24	amph-gabbro	cpx (1), amph (30), pl (61), mag (8), qz (tr), sulf (tr)	Layered fine-med-coarse-grained; trace of cpx and sulf; unzoned but inclusion and MI-rich pl
KS-17	ol-amph-gabbro	ol, cpx, amph, pl, mag, sulf (tr)	Medium-grained, well equlibrated; MI in ol, cpx and amph; incl of mag in plag
*Plutonic rocks*			
KS-3	ol-gabbro	ol (2), cpx (18), opx (3), pl (73), mag + ilm (4)	Lava with enclosed medium-grained plutonic fragment; normally-zoned px; incl-rich, oscillatory-zoned pl
KS-31	ol-amph-gabbro	ol (2), cpx (5), opx (3), amph (30), pl (58), mag + ilm (2)	Fine-medium-grained; Two types of cpx: cpx with amph halo, sometime enclosing ol, and cpx in resorption rims of amph; some ol with mag-rich symplectite rim; incl-rich pl
KS-22	ol-amph-gabbro	ol (2), cpx (14), opx (1), amph (10), pl (70), mag + ilm (3), sulf (tr)	Fine-medium-grained; trace opx; ol and cpx surrounded by amph; incl-rich and slightly zoned pl; mag and sulf usually together
KS-16	amph-gabbro	opx (3), amph (20), pl (74), mag + ilm (3), sulf (tr), ap (tr)	Medium-coarse-grained; bimodal pl population: anorthite and labradorite; pl has normal and reverse zoning; hbl oikocrysts
KS-4	amph-gabbro	cpx (1), opx (4), amph (17), pl (74), mag + ilm (3), qz (0.6)	Fine-grained; px rimmed by amph; zoned and incl-rich pl
KS-6	amph-gabbro	opx (2), amph (28), pl (66), mag + ilm (4)	Coarse-grained; euhedral to subhedral amph; pl has patchy and normal zoning zoning; MI in pl and amph

*ol* olivine, *pl* plagioclase, *amph* amphibole, *cpx* clinopyroxene, *opx* orthopyroxene, *sulf* Sulphide, *qz* quartz, *mag* magnetite, *ilm* ilmenite, *bio* biotite, *ap* apatite, *MI* melt inclusion, *incl* inclusion

Thin sections were imaged by Hitachi S-3500N scanning electron microscope (SEM) using backscattered electrons (BSE) obtained at 15 or 20 kV. Major element analyses were performed on a five-spectrometer Cameca SX-100 electron microprobe, calibrated on a variety of oxide and mineral standards. Analytical conditions were 15 or 20 kV acceleration voltage, and 10 nA focused beam for crystals. To minimize alkali loss during analyses of hydrous melt inclusions (MI) and interstitial glass the beam current was dropped to 4 nA and beam diameter increased to10 μm. Ferric iron contents were estimated using the stoichiometric methods of Droop (1987) for spinel, Wood and Banno (1973) for clinopyroxene, and Holland and Blundy (1994) for amphibole.

Volatile contents and some trace elements in glassy melt inclusions within plagioclase, orthopyroxene, hornblende, magnetite and ilmenite crystals, were analysed by secondary ion mass spectrometry (SIMS) at the NERC ion-microprobe facility, University of Edinburgh, using a Cameca IMS-4f instrument. Analyses were performed with a nominal 10 kV primary beam of O^−^ ions and 5 nA beam current focused to a ~ 20 µm diameter spot at the sample surface. H_2_O was measured as ^1^H^+^ secondary ions at a nominal mass resolving power (*M*/∆*M*) of 300 and 25 µm image field. Some trace elements were analysed simultaneously with ^1^H^+^. For CO_2_, measured as ^12^C^+^, a higher *M*/∆*M* = 800–1000 was applied to resolve ^24^Mg^2+^ from ^12^C^+^, and a 20 µm image field used. Positive secondary ions were extracted at 4.5 kV with an offset of 50 V (for C) and 75 V (for H) to reduce transmission of molecular ions. Pumping to a vacuum of ≤ 10^−9^ Torr minimized the background to ~ 2 counts per second for ^12^C and ~ 300 cps for ^1^H. Minimum detection limits (calculated from 3 s.d. on backgrounds) were ~ 11 ppm CO_2_ and ~ 70 ppm H_2_O. We calibrated H_2_O and CO_2_ against synthetic basaltic glass standards (Lesne et al. 2011) containing 0 to 3 wt% H_2_O and 0–2000 ppm CO_2_. Working curves of ^1^H/^30^Si vs H_2_O and ^12^C/^30^Si vs CO_2_ gave straight lines with *R* ^2^ ≥ 0.99.

### Experiments

The objective of the high pressure and temperature experiments was to investigate the differentiation conditions that led to the observed geochemical diversity in lavas and xenoliths. The most magnesian (primitive) basalts on St. Kitts have up to 7 wt% MgO and phenocrysts of plagioclase, olivine (Fo_<70_) and clinopyroxene (Toothill et al. 2007). Fractional crystallisation of these phases from such basalts can produce St. Kitts’ lower MgO basalts and basaltic andesites (Toothill et al. 2007). Orthopyroxene and Fe–Ti oxide phenocrysts do not appear until the host lava is basaltic andesite with ~ 4 wt% MgO. Given the presence of orthopyroxene in many St. Kitts xenoliths, we chose an experimental starting material with slightly more evolved composition than the most MgO-rich St. Kitts basalt. In this way, we increased the likelihood of attaining multiple saturation with a variety of minerals observed in xenoliths, and generating sufficiently large pools of experimental melt to analyse by electron microprobe. Conversely, the abundance of liquidus olivine was reduced.

The selected starting composition (K56) is basaltic andesite lava from Black Rocks with microphenocrysts of plagioclase (28 vol%), olivine (1.5 vol%), oxides (1 vol%) and traces of clinopyroxene and orthopyroxene (Baker 1984). The sample KS_BR1 used in this study is identical to K56 and was kindly provided by Rob Watts. The major element compositions of K56 and KS_BRl are presented in Table 2. K56 is chemically similar to a basaltic andesite from Mont Pelée, Martinique (031-22b1; Table 2) studied experimentally by Pichavant et al. (2002a, b), allowing the two sets of experimental results to be considered together.

**Table 2:** Experimental starting composition

	K56^a^	KS_BRl^b^	031-22b^c^
SiO2	53.77	54.64	53.00
TiO2	0.96	0.95	0.78
Al2O3	18.24	18.35	19.00
Cr2O3	0.00	0.03	–
FeO*	9.18	8.63	8.85
MnO	0.22	0.25	0.17
MgO	3.82	3.94	4.24
CaO	8.68	8.55	9.60
Na2O	3.56	3.54	2.79
K2O	0.44	0.49	0.67
P2O5	0.12	0.08	0.11
NiO	0.00	0.00	–
Total	98.99	99.44	99.78
	Mg# 43	Mg# 45	Mg# 46

FeO* is iron total

^a^Basaltic andesite from Black Rocks, St Kitts (Baker 1984)

^b^Basaltic andesite starting composition used in this study

^c^Martinique basaltic andesite starting composition of Pichavant et al. (2002a, b)

Powdered KS_BRl was dried at 100 °C for 4 h and then fused in a 1 atm gas mixing furnace at *f*O_2_ = NNO + 1 log unit in a platinum crucible. Two cycles of melting (2 and 4 h duration) and grinding were carried out to produce a chemically homogeneous glass, as determined by electron microprobe (Table 2). Gold capsules of 2.5–3.0 mm OD were filled with the crushed glass (15–20 μm grain size), to which volatiles were added as H_2_O ± Ag_2_C_2_O_4_ to create three different starting materials with the following initial molar fractions of H_2_O/(H_2_O + CO_2_): *X*H_2_O = 1, 0.66 and 0.33. The total added volatile content in each experiment was 9.5–10 wt%.

Experiments were carried out at 2.4 kbar in internally heated vessels at Université d’Orleans pressurized with Ar–H_2_ mixtures (Pichavant and Macdonald 2007). Temperature was measured with two S-type thermocouples with uncertainty ± 5 °C. The thermal gradient for a 3 cm-long capsule was < 5 °C. Run duration, following Pichavant et al. (2002a, b), was between 22 and 6 h. Three experimental capsules, plus redox sensor capsule, were placed together in a thin alumina tube held by a Pt wire at the furnace hot spot. The wire was fused electrically at the end of an experiment to achieve isobaric drop-quench at ~ 100 °C/s. Experiments were performed at a hydrogen fugacity (*f*H_2_) corresponding to NNO + 1. *f*H_2_ and *f*O_2_ are related via the dissociation of H_2_O:

1 $${\text{H}}_{ 2} {\text{O}} = {\text{H}}_{ 2} + {\raise0.5ex\hbox{$\scriptstyle 1$} \kern-0.1em/\kern-0.15em \lower0.25ex\hbox{$\scriptstyle 2$}}{\text{O}}_{ 2} .$$

An Ni–Pd *f*O_2_ sensor (e.g. Scaillet et al. 1995; Pichavant and Macdonald 2007) was used to measure *f*O_2_ during each run. The sensor was composed of two pellets of NiPd alloys plus Ni metal to give different initial Ni/Pd ratios. The pellets were loaded into a Pt capsule together with excess H_2_O. The metal phase was analysed after the experiment to calculate *f*O_2_ of the sensor. For any individual charge that is H_2_O-undersaturated (*a*H_2_O < 1) *f*O_2_ differs from that of the sensor by 2log *a*H_2_O, according to Eq. (1). We calculated *a*H_2_O for each charge using the method of Burnham (1979) at the measured (or estimated) H_2_O content of the glass (see below). Experimental *f*O_2_ was then calculated from *a*H_2_O and *f*H_2_.

Quenched experimental charges were imaged by SEM and analysed for major elements by Cameca SX100 and JEOL JXA8530F electron microprobes. Larger glass pools and metallic sensors were analysed on the SX100, using the same analytical procedure as for natural samples. Run product crystals and smaller glass pools were analysed on the JXA8530F. Typical analytical conditions for minerals were 10 kV, 10 nA and 100 nm beam size; for glasses 10 kV, 2 nA and10 μm beam size. In experiments with *a*H2O < 1 glass pools were not big enough to analyse with a defocused beam and beam size was reduced to 4 μm; for some glasses (Runs 3–3 and 4–2) we were obliged to use a focused beam, leading to significant alkali loss (< 37% relative, Table 3). Modal proportions of phases were obtained by mass balance calculations and presented in Table 3.

**Table 3:** Experimental run conditions and results

Run No	*X*H_2_O^init^	H_2_O* (wt%)	*a*H_2_O	log *f*O_2_, bars	DNNO, bars	Phase assemblage and proportions (wt%)	$\sum R^{ 2}$	Na_2_O lost^§^	H_2_O SIMS wt%	CO_2_ SIMS ppm	*X*H_2_O^final^	H_2_O^†^ wt%	CO_2_^†^ ppm
Run 1, 2.4 kbar, 1025 °C, *16* *h*, XNi = 0.64													
#1	1	9.5	0.90	− 9.6	− 0.3	Glass (100)			5.9 ± 0.2	16 ± 1	1	5.97	–
#2	0.66	3.8	0.44	− 10.3	0.3	Gl (57), ol (3), opx (tr), cpx (7), pl (31), mg (1)	0.18	0	3.8 ± 0.1	813 ± 10	0.49	3.29	870
#3	0.33	1.5	0.20	− 10.9	1.0	Gl (33), cpx (16), opx (tr), pl (47), mg (3)	0.70	24			0.23	1.96	1460
Run 2, 2.4 kbar, 1000 °C, *22* *h*, XNi = 0.67													
#1	1	9.8	0.89	− 10.1	− 0.2	Glass (98), cpx (1), mg (1)	0.09	0	6.0 ± 0.2	16 ± 11	1	5.98	–
#2	0.66	3.9	0.48	− 10.6	0.3	Gl (41), cpx (10), opx (9), pl (37), mg (3)	0.98	22			0.56	3.64	790
#3	0.33	1.5	0.22	− 11.3	1.0	Gl (27), cpx (23), opx (tr), pl (47), mg (2)	0.82	18			0.25	2.10	990
Run 3, 2.4 kbar, 975 °C, *22* *h*, XNi = 0.41													
#1	1	9.5	0.90	− 9.8	− 0.1	Glass (68), amph (12), cpx (5), pl (13), mg (2)	0.33	10	6.3 ± 0.1	30 ± 26	1	6.21	–
#2	0.66	3.6	0.47	− 10.3	0.0	Glass (36), *amph* (20), cpx (5), pl (35), mg (4)	0.42	28			0.56	3.69	1070
#3	0.33	1.3	*0.20*	− 11.1	− 0.5	*Glass, pl, cpx, opx, sp*					*0.25*	*2.09*	*960*
Run 4, 2.4 kbar, 950 °C, 12 h, XNi = 0.36													
#1	1	10	0.90	− 10.0	0.0	Glass (59), amph (21), pl (18), mg (2)	0.10	6			1	6.32	–
#2	0.66	3.9	0.45	− 10.6	0.8	Glass (42), amph (33), pl (24), mg (tr)	0.84	37			0.54	3.57	970
#3	0.33	1.8	*0.20*	− 11.3	− 0.2	*Glass, pl, cpx, opx, ilmenite, sp*					*0.25*	*2.08*	*940*

Phase assemblages in italics were analysed by SEM

*a*H_2_O—calculated from H_2_O† melt using the model of Burnham (1979)

log*f*O_2_—calculated from XNi in sensor and *a*H_2_O at P & T of experiment (Pownceby and O’Neill 1994)

*X*H_2_O^init^—initial molar fraction H_2_O in capsule

*X*H_2_O^final—^final molar fraction H_2_O in fluid (values in italics are estimates)

^§^—wt% Na_2_O lost based on mass balance

XNi—molar fraction Ni in NiPd sensor

* H_2_O added to the capsule

^†^ Calculated dissolved H_2_O and CO_2_ at run conditions using MagmaSat (Ghiorso and Gualda 2015)

## Results

### Petrography

Based on petrographic observations of 40 thin sections (Table A3 Supplementary) we subdivided the xenoliths into *cumulates*, with relatively high variance mineral assemblages that represent *instantaneous solid compositions* (Morse 1976), and *plutonics*, whose texture and composition is consistent with complete solidification of an aliquot of crystal-rich magma during cooling to the solidus [i.e. total solid composition of Morse (1976)]. This bipartite distinction is used throughout the paper. Note that the bulk compositions of cumulates are not equivalent to any magma type (Arculus and Wills 1980); their nomenclature follows Wager et al. (1960).

Xenolith mineralogy is dominated by calcic plagioclase and hornblende. Minor olivine (≤ 5 vol%) is widespread, but is frequently observed enclosed by pyroxene and/or amphibole. An important feature of the St. Kitts xenoliths is the presence of two pyroxenes in more than half of the collected samples, although orthopyroxene is more common in plutonics than in cumulates. Oxide minerals occur in all samples with the exception of cumulate xenolith KS-8. Nearly half of the analysed xenoliths contain co-existing ilmenite and magnetite, although ilmenite is confined to plutonic varieties. Plutonic olivine-gabbro xenolith KS-3 is unique: it has exsolved Fe–Ti oxide pairs, with single grains showing discrete ilmenite–magnetite lamellae. Rare biotite was found in a single plutonic xenolith (KS-14). Quartz was found in five plutonic samples, although it is not always easy to distinguish igneous quartz from xenocrystic/inherited quartz phenocrysts. Sulphide is a widespread accessory phase. Melt inclusions are common and were found in amphibole, orthopyroxene, plagioclase, and oxides.

The relative crystallisation order of plutonic and cumulate xenoliths, determined from textural observations, shows a consistent pattern. Olivine, when present, is always the first phase to crystallise, with the sole exception of an olivine-norite (KS-11) sample where magnetite precedes olivine. Elsewhere, magnetite is the next phase to crystallise and occurs throughout the crystallisation sequence of all xenoliths. The order of orthopyroxene and clinopyroxene appearance varies: they either co-crystallise or clinopyroxene precedes orthopyroxene, which may reflect slight differences in crystallisation temperature between the samples (e.g. Leuthold et al. 2014, Fig. 19). Unusually, in KS-3 clinopyroxene crystallises after orthopyroxene. Plagioclase never crystallises before pyroxenes and typically appears either prior to amphibole or co-crystallises with it. There are two xenoliths, KS-22 and KS-6, in which amphibole crystallised before plagioclase. Amphibole often demonstrates a two-stage crystallisation: early crystallisation alongside pyroxene or plagioclase, and late-stage, interstitial crystallisation. Where present, biotite, apatite and quartz are always last in the crystallisation sequence. Sulphide occurs only as inclusions in magnetite. Ilmenite in plutonic xenoliths co-crystallises with late-stage magnetite.

**Fig. 2:**
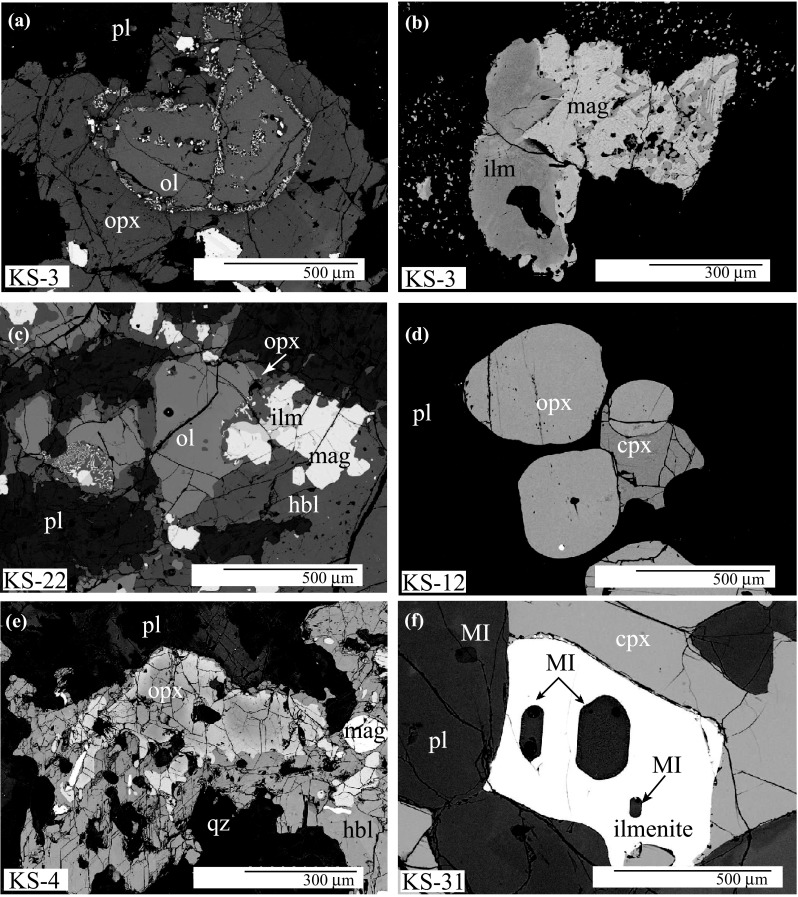
BSE images of representative textures of xenoliths in Table 1. **a** Development of orthopyroxene–magnesioferrite oxidation symplectites within olivine grain (KS-3). **b** Magnetite–ilmenite exsolution (KS-3), **c** Olivine oikocrysts with orthopyroxene reaction rim (KS-22). **d** Chadacrysts of orthopyroxene and clinopyroxene in plagioclase (KS-12). **e** Zoned orthopyroxene with MgO-rich core in poikilitic amphibole (KS-4). **f** Silica-rich melt inclusions in ilmenite (KS-31)

**Fig. 3:**
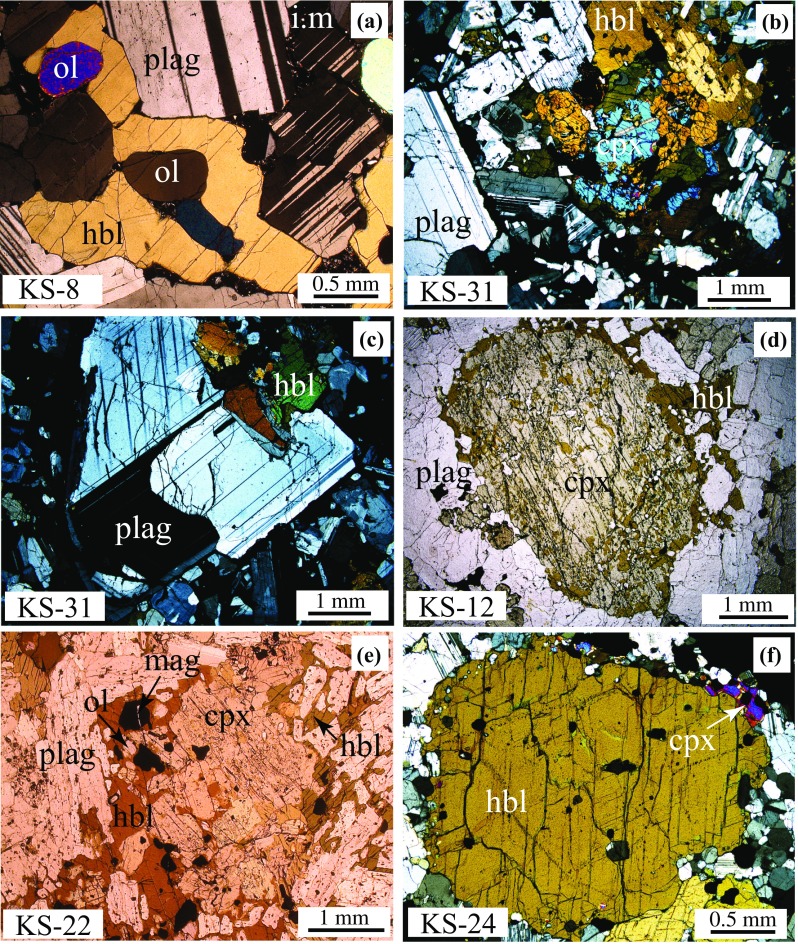
Photomicrographs of representative xenolith textures (Table 1) in plane-polarised light (ppl) and cross-polarised light (xpl). **a** Olivine-amphibole gabbro (KS-8) showing orthocumulate texture (ppl). **b**, **c** Olivine-amphibole gabbronorite (KS-31) displaying several stages of amphibole crystallisation (xpl); **b** Clinopyroxene replacement by amphibole. Note clear clinopyroxene twinning and normal plagioclase zoning, **c** amphibole crystallisation prior to plagioclase. Plagioclase shows resorption rim with melt inclusions. **d** Clinopyroxene grain exhibiting reaction to amphibole around the rim and interstitial amphibole (olivine-amphibole gabbronorite, KS-12) (ppl). **e** Similar texture in olivine-amphibole gabbro, KS-22 (ppl). **f** Clinopyroxene halo around phenocrystic amphibole (amphibole gabbro, KS-24) (xpl)

Plutonic and cumulate xenolith types display striking variation in modal proportion of minerals, textures and amount of interstitial glass. A key difference between the two types is the strong mineral zoning observed in plutonic xenoliths (Fig. 2e). Textures suggestive of mineral–mineral and mineral-melt reactions, such as symplectic and poikilitic textures, as well interstitial amphibole (Fig. 3e), are more common in plutonic xenoliths. In contrast, cumulate xenoliths are minimally zoned, with adcumulate textures and euhedral crystals (Figs. 2f and 3a). Based on textural observations and modes, plutonic and cumulate xenoliths were subdivided into seven diagnostic rock types; note that amphibole gabbros and olivine-amphibole gabbros occur as both plutonic and cumulate xenolith types.

#### Plutonic xenoliths

*Amphibole gabbros* are hypidiomorphic granular (1–2 mm grain size) with fabrics that range from well-foliated to isotropic. Amphibole occurs as both subhedral and intergranular crystals. Subhedral crystals define the foliation where present (e.g. KS-6). Intergranular amphiboles contains inclusions of plagioclase in their cores and orthopyroxene in their rims. Oxides occur as inclusions in plagioclase and amphibole but also form intergranular crystals in some samples (KS-16) with inclusions of plagioclase, amphibole and Sulphide. There is widespread evidence of amphibole–pyroxene reaction. In some samples, amphibole rims contain abundant orthopyroxene inclusions; in others, amphibole forms reaction rims around orthopyroxene and clinopyroxene. Plagioclase is strongly zoned with both concentric and patchy variants. Large plagioclase grains commonly contain inclusions of amphibole. In some samples (KS-4, KS-16) plagioclase (± orthopyroxene) forms a mortar texture composed of small, interlocking grains around larger crystals of amphibole and zoned plagioclase. Interstitial quartz may be associated with small plagioclase crystals. Melt inclusions are common.

*Olivine amphibole gabbros* are isotropic, hypidiomorphic granular, with grain size ≤ 0.7 mm. Orthopyroxene mainly forms reaction rims around olivine (Fig. 2c), typically as symplectite intergrowths with sub-micron opaque oxides. Amphibole reaction rims around clinopyroxene grains are common (Fig. 3b) and there is persuasive textural evidence for infiltration of amphibole-forming fluids along grain boundaries. Clinopyroxene often has sieve textures with abundant melt inclusions. Plagioclase is euhedral with normal zoning and often intergrown with amphibole (Fig. 3c). Oxides are either anhedral inclusions in amphibole or form subhedral grains up to 0.8 mm (Fig. 2c). Ilmenite forms euhedral and anhedral grains, although it may also be interstitial. Small, glassy melt inclusions are ubiquitous and present in all minerals apart from olivine (Fig. 2f).

*Olivine gabbro*, as represented by KS-3, is a texturally complex, amphibole-free gabbro in contact with the host lava. Lava and xenolith display similar mineralogy. The xenolith is crossed by fractures that are filled with microcrystals of oxides, anhedral orthopyroxene and plagioclase. In the xenolith part of the sample orthopyroxene forms reaction rims around partially iddingstised olivine (Fig. 2a), similar to those in olivine-amphibole gabbros. Clinopyroxene forms large subhedral phenocrysts (≤ 2 mm) with chadacrysts of magnetite. Some clinopyroxene is consumed in plagioclase-forming reactions. Plagioclase has strong normal zoning and often displays resorption rims containing small melt inclusions. Intergrown magnetite–ilmenite pairs show exsolution textures, suggestive of slow cooling (Fig. 2b).

#### Cumulate xenoliths

*Amphibole gabbro* is a well-equilibrated, layered adcumulate (KS-24, Fig. 3e) made up of two distinct layers with similar mineralogy. The coarse layer comprises euhedral amphibole (≤ 3 mm) and plagioclase (≤ 2 mm), and subhedral magnetite (≤ 2 mm). Large plagioclase grains are moderately zoned with one or more resorption rims containing abundant melt inclusions. The same minerals in the fine-grained layer have a mortar texture with grain size less than 0.3 mm around a few relict larger crystals with similar textures to the coarser layer, suggesting partial recrystallisation. Clinopyroxene forms small subhedral crystals with grain size < 0.2 mm and may also form halos around amphibole phenocrysts (e.g. KS-24; Fig. 3f).

*Olivine amphibole gabbros* can be subdivided texturally into mesocumulate and orthocumulate variants. Orthocumulates (KS-8, KS-15, KS-21) comprise large interlocking amphibole and large euhedral plagioclase 1–5 mm across. Olivine forms chadacrysts in amphibole (Fig. 3a) and is usually fresh apart from occasional iddingsitised grains in KS-15. Minerals in KS-8 and KS-15 are well equilibrated and rarely zoned. In KS-15 abundant magnetite forms large (1–3 mm) euhedral crystals, whereas KS-8 is distinguished by a lack of oxides. Medium-grained mesocumulate (KS-7) contains a small amphibole-rich, oxide-free xenolith enclave with mineral compositions similar to those of the host xenolith. Amphibole is poikilitic with chadacrysts of clinopyroxene, plagioclase, olivine and Al-rich magnetite. Magnetites range from large interstitial grains (≤ 1.5 mm diameter) to small euhedral grains of ≤ 200 μm. Both variants contain pockets of interstitial microvesicular glass with microlites of clinopyroxene and plagioclase.

*Olivine norite* (KS-11) is dominated by sub-euhedral adcumulate plagioclase, magnetite and olivine with well-equilibrated 120º grain boundaries. Poikilitic orthopyroxene is interstitial to plagioclase. There are additionally a few larger, anhedral orthopyroxenes with grain size ≤ 2 mm. Olivine is iddingsite-free and contains glassy melt inclusions. Plagioclase twin planes have some slight flexure, suggestive of deformation.

*Olivine amphibole gabbronorite* (KS-12) is a texturally complex, transitional type of xenolith. It displays cumulative textures, but with strongly zoned, altered minerals and although grouped with cumulates equally be described as plutonic. Euhedral plagioclase and clinopyroxene are enveloped by poikilitic amphibole. Clinopyroxene grains commonly show reaction to amphibole along grain boundaries and fractures (Fig. 3d). Conversely, some anhedral amphiboles are mantled by complex coronas of intergrown clinopyroxene and plagioclase. Magnetite comprises anhedral blebs and subhedral crystals, both as inclusions in silicate phases and interstitial grains.

### Mineral and glass chemistry

Representative mineral analyses are presented in Table A1 (Supplementary). For pyroxene and amphibole, Mg# is expressed as ${\text{Mg}}/\left( {{\text{Mg}} + \sum {\text{Fe}}} \right)$, where $\sum {\text{Fe}}$ denotes total iron. Sulphides are present in many St. Kitts xenoliths, but are too small to be analysed. There is a relatively wide variation in amphibole and plagioclase mineral composition across the different xenolith types, but relatively limited chemical variation in pyroxenes, spinel and olivine.

**Fig. 4:**
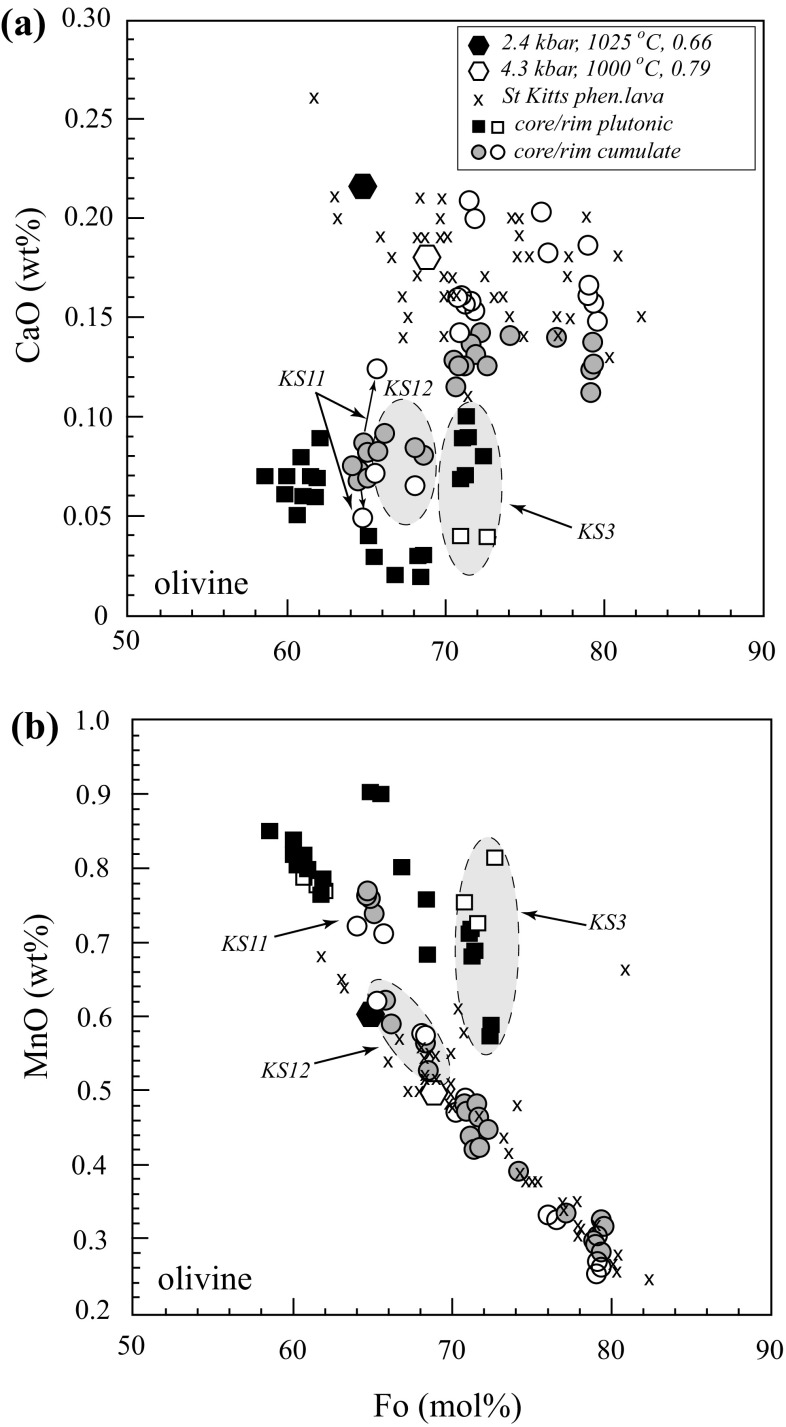
Olivine compositions in terms of wt% **a** CaO and **b** MnO concentration from lavas, experiments and xenoliths as a function of forsterite (Fo) content. Olivine phenocrysts from St Kitts lavas are from Toothill et al. (2007); cumulate and plutonic xenoliths from this study (selected samples are labelled). Black and white diamonds are experimental run products from this study (Run1#2) and HAB7 of Pichavant et al. (2002a, b) at the given P, T and *X*H_2_O

*Olivine* is well preserved, rarely iddingsitized (the exception is KS-15), and ranges in composition from Fo_77_ to Fo_59_. Olivine from cumulate xenoliths tends to have higher Fo. The range in xenolith olivine is comparable to that of phenocrysts in St. Kitts lavas (Fo_82–62_; Fig. 4). Individual xenolith grains are typically homogeneous (≤ 4 mol% variation in Fo). The greatest range is found in plutonic varieties (Figs. 1 and 4) where olivine is rimmed by orthopyroxene-oxide symplectites (Fig. 2a, c). Similar textures have been attributed by Johnston and Stout (1984) to oxidation of olivine.

In terms of minor components, NiO is consistently ≤ 0.07 wt%; CaO varies between 0.01 and 0.26 wt% (Fig. 4). There is no clear correlation between Fo and CaO or NiO. The majority of olivine in cumulates overlap the CaO contents (0.15–0.22 wt%) of phenocrysts in lavas (Fig. 4a). Conversely, the CaO contents in plutonic olivine tend to be much lower (0.01–0.11 wt%), although there are two cumulate xenoliths, KS-12 and KS-11 with olivine Fo_≤70_ and < 0.10 wt% CaO, akin to plutonics (Fig. 4a). MnO ranges from 0.2 to 0.9 wt%, and is negatively correlated with Fo (Fig. 4b). MnO contents in cumulate olivine are very similar to those from lavas, whereas plutonic olivine is displaced to higher MnO. Unlike CaO, MnO in KS11 and KS12 olivine does not deviate from the rest of the cumulates.

*Oxides* are ubiquitous in St. Kitts xenoliths, reaching 12 vol% in KS-15. Only olivine-amphibole gabbro KS-8 is oxide-free. Oxides occur as inclusions in silicate phases, along grain boundaries, in interstitial melt and inside some melt inclusions. Individual euhedral oxides may be up to 1.5 mm (Fig. 2f).

**Fig. 5:**
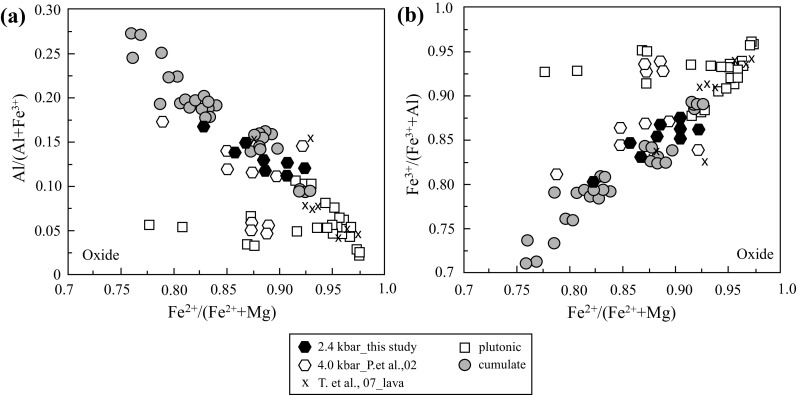
Spinel compositions, expressed in terms of Al# (**a**) and Fe^3+^# (**b**) versus Fe^2+^# from St. Kitts lavas (T. et al. 07—Toothill et al. 2007), cumulate and plutonic xenoliths (this study) compared with experimental spinel from Pichavant et al. 2002a, b (P.et al. 02)

The dominant oxide is magnetite-rich spinel, with relatively high TiO_2_ (4–14 wt%) except for two Ti-poor spinel grains from KS3 (Table A2 Supplementary). TiO_2_-rich magnetite has also been reported from St. Kitts lavas (Toothill et al. 2007). Cr_2_O_3_ contents are consistently low (≤ 0.4 wt%; Table A2 Supplementary). There are three distinct groupings of spinel composition in terms of Al# (= Al/(Al + Fe^3+^)) and Fe^3+^# (= Fe^3+^/(Fe^3+^+Al)) versus Fe^2+^/(Fe^2+^+Mg) (Fig. 5). The two dominant groupings lie along the magnetite (Fe_3_O_4_)–spinel (MgAl_2_O_4_) exchange vector: Al-rich magnetite occurs in cumulates, whereas Al-poor magnetite, coexisting with ilmenite, occurs in plutonics (Table A2 Supplementary, Fig. 5a). Phenocrysts from lavas overlap with plutonic spinel at the low-Al end of the trend. Low-Al spinels from plutonics (notably KS-3) show a subsidiary trend to lower Fe^2+^/(Fe^2+^+Mg), consistent with the magnetite–magnesioferrite (MgFe_2_O_4_) exchange vector. This group is found in plutonic xenoliths with olivine breakdown symplectites (Fig. 2a), although individual spinel grains within the symplectites are too small to analyse. Johnston and Stout (1984) recognized a significant magnesioferrite component in spinel associated with oxidation-related symplectites around olivine.

*Clinopyroxene* is present in three cumulate and four plutonic xenoliths with modal proportions from 0.8 to 18 wt%. Texturally, clinopyroxene can be subdivided into the following groups: homogeneous crystals typical of cumulates (Fig. 2d); normally zoned clinopyroxene in plutonics with diopside cores and augite rims (KS-4); and clinopyroxene with non-systematic sectoral compositional variations. The latter are Al- and Ca-rich diopside and augite that are also high in Fe^3+^/ΣFe (as calculated from stoichiometry), ranging from 0.1 to 0.5.

**Fig. 6:**
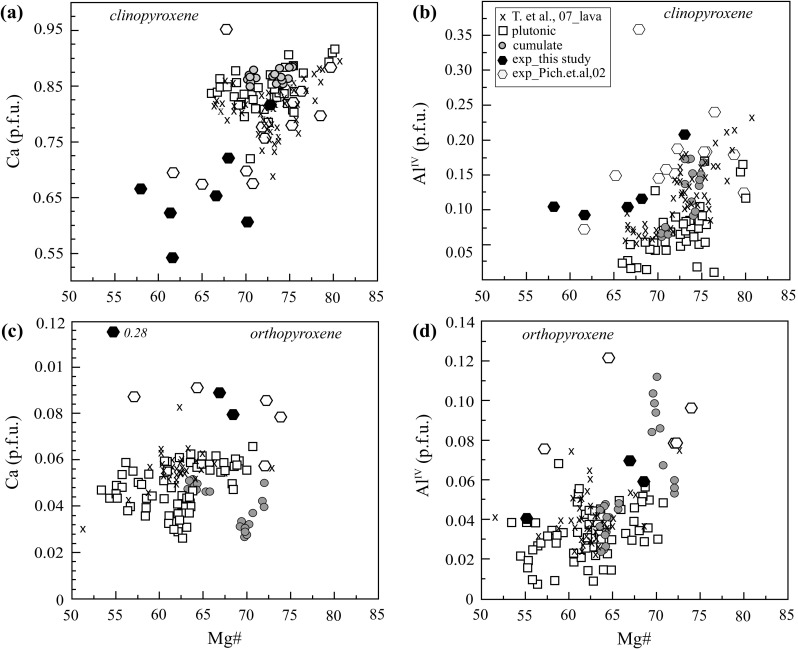
Clinopyroxene (**a**, **b**) and orthopyroxene (**c**, **d**) compositions from lava phenocrysts (Toothill et al. 2007), xenoliths and experiments in terms of Ca (**a**, **c**) and Al^iv^ (**b**, **d**), expressed as cations per formula unit, versus Mg#. Note high-Ca and low-Ca trends for clinopyroxene phenocrysts from lavas (**a**). A single experimental pigeonite with high Ca is labelled

Clinopyroxene phenocrysts from lavas and plutonic xenolith have a wide range of Mg# (69–80) with a relatively small array of Ca (0.66–0.86 apfu; Fig. 6a). The range of Mg# in clinopyroxene from xenoliths is muted (70–75). Both Ca and tetrahedral aluminium (Al^iv^) decrease with decreasing Mg# (Fig. 6a, b), although the Al^iv^ decrease is non-linear, with an abrupt drop at Mg# of 75. In contrast to olivine and spinel, there is no systematic difference between plutonic and cumulate clinopyroxenes and both varieties overlap with phenocrysts from lavas. There is a small increase of TiO_2_ (0.3–1.0) with increasing Mg#. TiO_2_ content in KS-7 is notably higher than all other clinopyroxenes (1.5–1.8 wt%).

*Orthopyroxene* is common in St. Kitts cumulates with modal abundances ≤ 12%. This is in contrast to xenoliths found on the southern islands of Grenada and St. Vincent, where orthopyroxene is common in andesite and dacite lavas, but lacking in xenoliths (Arculus and Wills 1980; Tollan et al. 2012; Stamper et al. 2014). Texturally, orthopyroxene can be divided into five groups: homogeneous phenocrysts (Fig. 2d); normally zoned chadacrysts in amphibole (KS-4, Table A2 Supplementary, Fig. 2e); phenocrysts with non-systematic sectoral zoning similar to clinopyroxene; poikilitic orthopyroxene (KS-11); and orthopyroxene–magnesioferrite symplectites (Fig. 2a). Orthopyroxene composition ranges between En_50_ and En_69_, with Wo_≤3_ (Table A2 Supplementary). Tetrahedral aluminium (Al^IV^) contents are low (0.005–0.12 apfu). There is a positive correlation between Mg# and Al^IV^ in xenolith orthopyroxenes (Fig. 6d), but no correlation between Ca content (0.03–0.07 pfu) and Mg#. Poikilitic orthopyroxene from KS-11 shows a wide range of Al^IV^ at similar Mg#. Orthopyroxene in lavas is of similar composition to xenoliths, but with a more restricted Mg# range, 65–51.

**Fig. 7:**
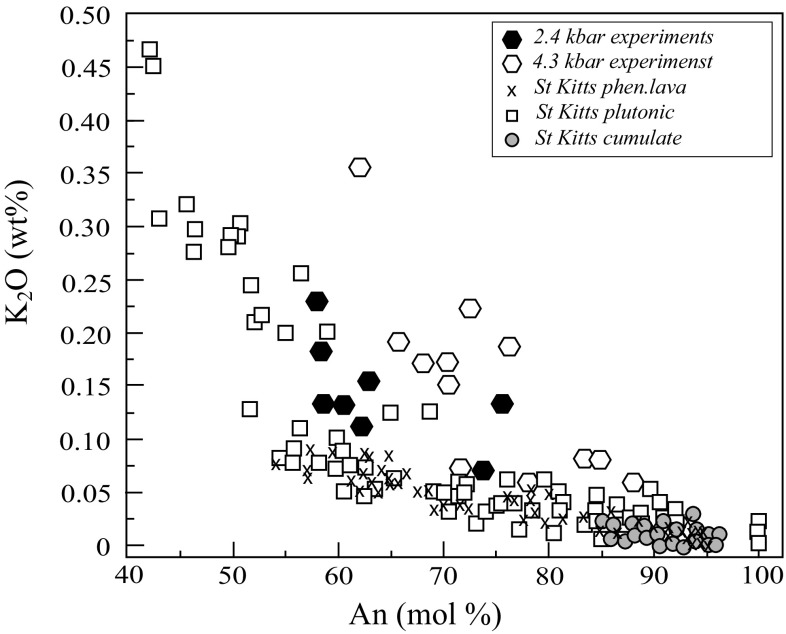
Plagioclase compositions, expressed as wt% K_2_O versus An content for lava phenocrysts (Toothill et al. 2007), xenoliths and experiments at 2.4 and 4 kbar

*Plagioclase* is modally dominant (≤ 80%) in all but one xenolith, KS-21 (Fig. 1a) and very calcic in composition, similar to other xenoliths from the Lesser Antilles (Baker 1968; Lewis 1973; Arculus and Wills 1980; Tollan et al. 2012; Cooper et al. 2017). However, St. Kitts plagioclase attains the most calcic compositions yet recorded, reaching almost pure anorthite (≤ 99.9 mol %) in two plutonic xenoliths (KS-16 and KS-3). There are five main textural varieties of plagioclase: (1) euhedral, high-An plagioclase without obvious zoning (e.g. KS-8, Fig. 3a and Table A2 Supplementary) or melt inclusions, confined to cumulate xenoliths; (2) plagioclase with calcic cores (An_>90_) separated abruptly from less calcic (An_<75_) rims with fine-scale oscillatory zoning (amplitudes of ± 10 mol % An) and abundant glassy melt inclusions (Fig. 3c); (3) normally-zoned plagioclase with almost monotonic decline from calcic core (An_>90_) to An_50_ rims, punctuated by occasional high amplitude (≤ 30 mol % An) calcic spikes; (4) plagioclase lacking a high-An core, but with patchy irregular zoning and tabular textures; (5) subhedral bytownite with irregular zoning. A striking difference between plutonic and cumulate xenoliths is the range in composition. Cumulates are characterised by a limited range in An (< 15 mol %), whereas plutonics may show exceptional ranges (≤ 50 mol % An), even within a single crystal, consistent with protracted in situ crystallisation. K_2_O concentrations in plagioclase range up to 0.14 wt%. Overall the variation in phenocryst compositions from lavas (core An_95_ to rim An_60_) and cumulates is less than in plutonic xenoliths (Fig. 7).

*Amphibole* is the second most abundant mineral in St. Kitts xenoliths, with modal proportion from 10 to 60% (Fig. 1a, Table 1), but is exceedingly rare in St. Kitts volcanics (Baker 1968; Toothill et al. 2007). Texturally, amphibole can be divided into two groups regardless of whether the xenolith is cumulate or plutonic. In the first group, amphibole forms an interlocking network of discrete, inclusion-poor grains, appearing to crystallise as a relatively late primocryst phase (Fig. 2c, e). Rarely, amphibole has a halo of clinopyroxene, suggestive of subsequent breakdown. This is especially evident where xenoliths are in direct contact with the host lava. (e.g. KS-24, Fig. 3f). In the second group, amphibole is an interstitial phase. In many samples, the interstitial amphiboles form large, optically continuous poikilocrysts, up to several mm across. Inclusions of olivine, oxides, clinopyroxene and plagioclase are common, and evidence of amphibole–clinopyroxene reaction is widespread around grain margins, along cleavage planes, or within poikilocryst interiors (Fig. 3d). This texture, which is widespread in cumulate xenoliths, is reminiscent of the distribution of residual melt, and suggestive of percolation of reactive hydrous melt or fluids through an anhydrous crystal mush, as documented in xenoliths from Martinique (Cooper et al. 2017), Grenada (Stamper et al. 2014) and the Solomon Islands (Smith 2014).

**Fig. 8:**
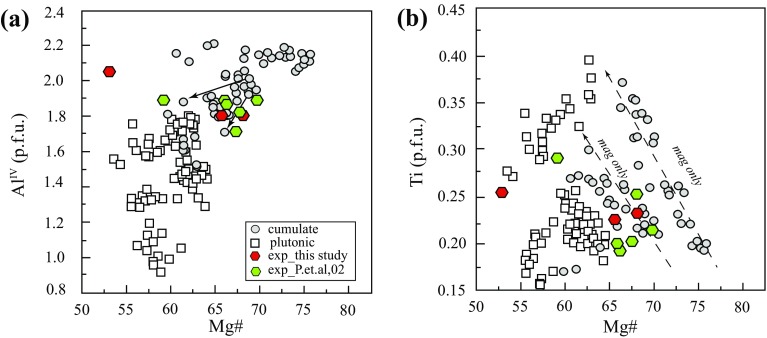
Amphibole compositions from St Kitts xenoliths and experiments expressed in terms of Al^iv^ and Ti, expressed as cations per formula unit, versus Mg#. Arrows on panel **a** show decrease in Al^iv^ from core to rim in KS-15. Ti content of amphibole **b** strongly depends on composition of co-crystallising oxides. Magnetite only (*mag only*) arrows show negative correlation of Ti and Mg# in cumulate xenoliths. Ilmenite is present only in plutonic xenoliths, giving rise to low-Ti amphiboles

According to the classification scheme of Leake et al. (1997, 2004), most St. Kitts amphibole is magnesiohastingsite with lesser tschermakite (KS-16, KS-31 and KS-4). Mg#, calculated with Fe total, is between 52 and 76. Subhedral and euhedral amphibole is normally zoned with < 5% variation in Mg#. There is no difference in amphibole composition between clinopyroxene-bearing and clinopyroxene-free xenoliths, although plutonic and cumulate amphibole differs markedly (Fig. 8a). Plutonic amphibole has lower Mg# (64–54, Fig. 8a) and Al^IV^ (≤ 1.8 apfu), whereas cumulate amphibole has higher Mg# (76–60) and higher Al^IV^ (1.6–2.2 apfu). Titanium contents are in the range 0.15 and 0.40 a.p.f.u. with trends of increasing or decreasing Ti with Mg# (Fig. 8b) according to the nature of the coexisting oxide mineralogy. Amphiboles on the increasing Ti trend come from xenolith that contain only magnetite (shown by arrows on Fig. 8b), whereas those on the decreasing trend come from samples with ilmenite. Viewed as a suite, amphibole trends resemble a fractionation sequence with Ti increasing to the point of ilmenite saturation and then decreasing.

*Glass* occurs as melt inclusions in crystals and as interstitial pockets, often vesiculated, in both xenolith varieties. Inclusions, ranging in size from ≤ 10 to 200 μm and from clear to brown in colour, are common in plagioclase and amphibole, and less so in olivine, pyroxenes and oxides. Inclusions often contain gas bubbles with typical volume fractions from 0 to 10%, and rarely 20–50% (Fig. 2f). Inclusions in olivine are usually devitrified or too small to analyse.

**Fig. 9:**
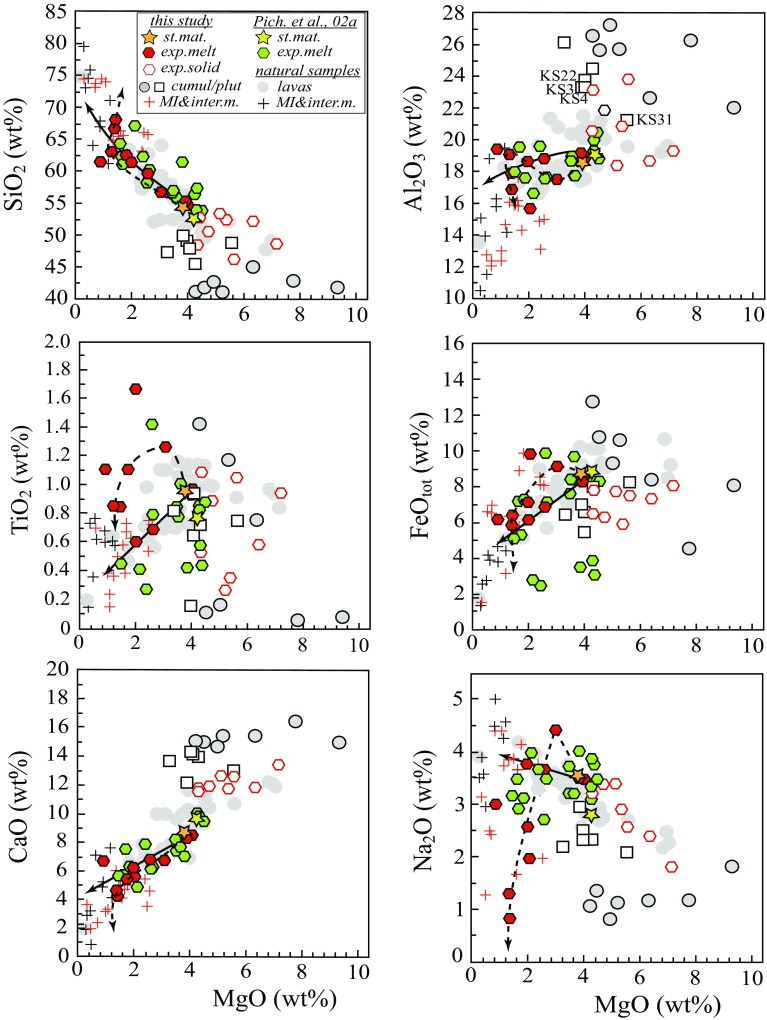
Chemical composition of experimental melts (*exp. melt*) and solid residues (*exp. solid*) from this study compared with bulk-rock major element variations in St Kitts lavas (Toothill et al. 2007; Turner et al. 1996; Baker 1984), melt inclusions in phenocrysts from cumulates (this study) and lavas (Toothill et al. 2007), and cumulate xenoliths (calculated from mineral modes and EMPA data). Experimental melts from Pichavant et al. 2002a, b (Pich. et al. 2002) also shown for comparison. *St.mat.* starting material, *cumul/plut* cumulate xenolith/plutonic xenolith, *MI&inter.m* melt inclusions and interstitial melt. Continuous and dashed lines illustrate melt evolution in experiments with *X*H_2_O = 1 and *X*H_2_O = 0.66, respectively. The trend to low Na_2_O in the latter experiments reflects, in part, Na loss during EMP analysis (Table 3)

Eleven melt inclusions and 13 interstitial glasses were selected for analysis (Table A2 and Table A4 Supplementary). Selected inclusions show no signs of post-entrapment leakage or devitrification. They range in composition from dacite to rhyolite (63.1–74.8 wt% SiO_2_ on an anhydrous basis) with no systematic correlation with the nature of the host crystal (Table A3 Supplementary and Fig. 9). Interstitial melt and melt inclusions are compositionally similar, although the former have lower H_2_O and, unusually, higher CO_2_ contents (Table A4 Supplementary). Melt inclusions from plutonic xenoliths are systematically more evolved (66.0–74.8 wt% SiO_2_) than those from cumulates, which are consistently andesitic (63.1–66.0 wt% SiO_2_). The occurrence of andesitic melt inclusions is surprisingly rare in global compilations of melt inclusions in volcanic rocks (Reubi and Blundy 2009). In fact, three melt inclusions with > 6 wt% H_2_O and 65–67 wt% SiO_2_ lie in the compositional gap shown by Reubi and Blundy (2009).

Overall, melt inclusions describe a fractionation trend from andesite to dacite (Fig. 9). Melt compositions overlap the silica-rich end of the whole–rock compositions of erupted lavas of St. Kitts and so provide information on the more evolved end of the liquid line of descent (Fig. 9). A striking feature of melt inclusions with < 3 wt% MgO is the trend to lower Na_2_O. All melt inclusions were analysed using a defocussed electron beam and, as there is no correlation between Na_2_O and H_2_O contents, we do not consider this to be an analytical artefact. Instead, in the absence of any Na-rich crystallising phase, this behaviour is suggestive of sequestration of Na_2_O into an exsolving volatile phase. In that case the Na_2_O maximum (~ 4.5 wt%) in whole-rocks and melt inclusions at around 3 wt% MgO would correspond to the onset of significant volatile exsolution.

**Fig. 10:**
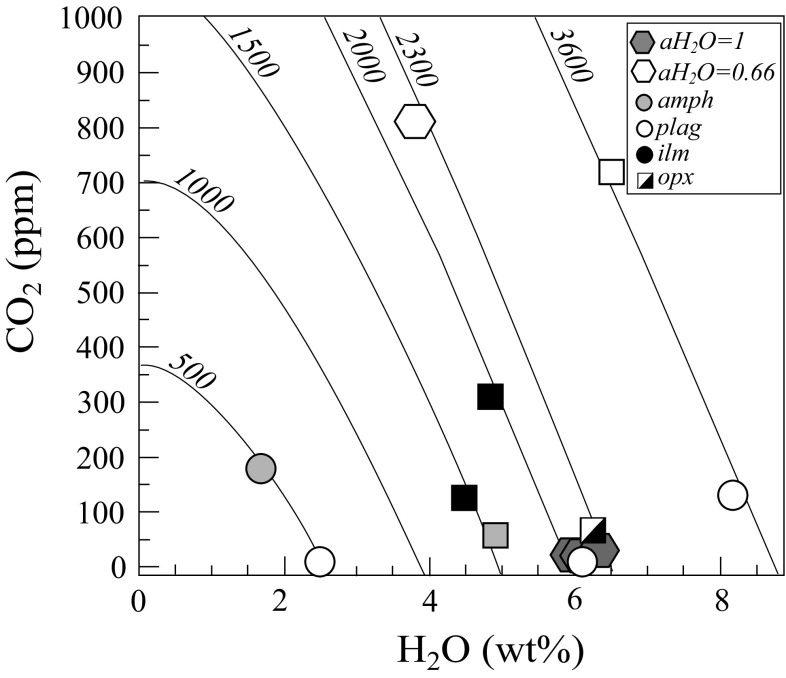
Volatile contents of melt inclusions and experimental glasses. Solid lines are illustrative isobars for generic basalt at 1000 °C after Newman and Lowenstern (2002)

Water content in melt inclusions, as measured by SIMS, ranges from 8.5 wt% to below detection. Melt inclusions in plagioclase (An_86–79_ and An_59_) show a broad range from 8.2 to 2.5 wt%, with the lower H_2_O associated with the less calcic hosts (Table A2 Supplementary, Fig. 10). CO_2_ contents range from below detection to over 1000 ppm, but do not correlate with H_2_O (Fig. 10). Notably, two interstitial glasses (not plotted on Fig. 10) from KS-15 have high CO_2_ (> 1000 ppm) but low H_2_O (< 1.6 wt%). Inclusions with lower H_2_O or CO_2_ are not associated with the bigger vapour bubbles. The high H_2_O content of many inclusions argues against significant H_2_O loss by diffusion through the host mineral, consistent with rapid pre-eruptive entrainment of the xenoliths in their host magma.

Chlorine contents are consistently high, reaching 3000 ppm in some melt inclusions (Table A3 Supplementary). Sulphur contents are low (< 350 ppm) consistent with the presence of sulphide minerals in many samples. There is no clear correlation between H_2_O and any other volatiles species. However, there is a positive correlation between sulphur and chlorine and CO_2_ indicative of degassing. Chlorine increases with decreasing MgO, consistent with the incompatible behaviour of Cl and limited partitioning of Cl into exsolving fluids (cf. Blundy et al. 2008).

### Intensive parameters of xenolith formation

#### Thermometry and oxybarometry

Studies of xenoliths from other Lesser Antilles islands (Cooper et al. 2017; Melekhova et al. 2015; Stamper et al. 2014; Tollan et al. 2012) show that T–P–*a*H_2_O–*f*O_2_ conditions under which xenoliths formed can vary widely, even for a single island. Nonetheless, the majority of xenoliths formed at pressures ≤ 4 kbar from magmas with initial H_2_O content between 2.5 and 4.5 wt%, and *f*O_2_ ranging from 0.5 to 4.5 log units above NNO. Here we use mineral and melt chemistry of St. Kitts xenoliths to constrain intensive parameters with the following oxythermobarometers (Table 4): amphibole–plagioclase thermometer of Holland and Blundy (1994), hornblende-liquid thermobarometer of Putirka (2016), and magnetite–ilmenite oxythermometers of Ghiorso and Evans (2008) and Andersen and Lindsley (1985). Note that amphibole–plagioclase thermometry is limited to plagioclase less calcic than An_90_ (Holland and Blundy 1994), a requirement met by only three xenoliths.

**Table 4:** Calculated intensive parameters for St. Kitts xenoliths

Sample	Type	*T* (°C)					P, kbar		ΔNNO	ΔNNO
		cpx–opx^a^	hbl-liq^b^	hbl-plag^c^	mag-ilm^d^	mag-ilm^e^	hbl-liq^b^	ol + sp + cpx + pl^f^	mag-ilm	mag-ilm
		(Put., 08)	(Put., 16)	(H&B,94)	(G&E,08)	(A&L,85)	(Put., 16)	(Z et al. 2017)	(A&L,85)	(G&E,08)
*Plutonic*										
KS-6	amph-gabbro		892	1027–915	798	836			0.9	1.0
KS-4	amph-gabbro	773–767		*782*	774–795	810			0.4	0.6
KS-16	amph-gabbro		885	*892*	821	840	4.0		1.2	0.8
KS-22	ol-amph-gabbro	880–935		*912*	843	810			0.1	− 0.1
KS-31	ol-amph-gabbro		813	*845*	830	810			0.9	0.6
KS-3	ol-gabbro	803–920			559	710–930			2.5/3.7	2.18
*Cumulate*										
KS-24	amph-gabbro			1022						
KS-12	ol-amph-gabbronorite	890–915	904	970			1.0			
KS-7	ol-amph-gabbro							6.4 ± 1.8		
KS15	ol-amph-gabbro		935				3.0			
KS-21	ol-amph-gabbro		968				3.6			
KS-17	ol-amph-gabbro							6.0 ± 1.2		

Italic represents minimum temperature calculated for least Ca-rich plagioclase in the sample

782 °C minimum temperature calculated for least Ca-rich plagioclase in the sample

^a^Clinopyroxene–orthopyroxene thermometer, Putirka 2008

^b^Hornblende-liquid thermobarometer, Putirka 2016

^c^Hornblende–plagioclase thermometer, Holland and Blundy 1994

^d^Magnetite–ilmenite oxythermometer Ghiorso and Evans 2008

^e^Magnetite–ilmenite oxythermometer, Andersen and Lindsley 1985

^f^Multiple reaction barometer, Ziberna et al. 2017; *ol* olivine, *sp* spinel, *cpx* clinopyroxene, *pl* plagioclase

With a few exceptions, we find good agreement between different thermometers (± 40 °C) applied to the same sample. Plutonic xenoliths generally record lower temperatures (890–770 °C) than cumulate xenoliths, consistent with their more complex textures and mineralogy and more evolved melt inclusions. The presence of exsolved Fe–Ti oxides in KS-3 (Fig. 2b) yields subsolidus temperatures suggestive of protracted cooling. Cumulate xenolith temperatures are in the range 1020–890 °C.

Oxygen fugacity (*f*O_2_) for plutonic xenoliths, calculated from coexisting Fe–Ti oxides, lies between NNO and NNO + 1 for all but KS3, which records a significantly higher *f*O_2_ (NNO + 2), consistent with the interpretation of symplectites around olivine (Fig. 2a) as products of oxidation, possibly associated with cooling. The calculated *f*O_2_ values for St. Kitts lavas estimated by Toothill et al. (2007) have a similar range, ΔNNO ± 1, to the un-oxidised plutonic xenoliths. It was not possible to calculate *f*O_2_ for the cumulate xenoliths due to lack of an appropriate assemblage.

#### Apatite saturation temperatures

The melt inclusions show a trend of decreasing P_2_O_5_ with increasing SiO_2_, consistent with apatite saturation. We have used the algorithm of Harrison and Watson (1983) to calculate apatite saturation temperatures for each melt inclusion. Values range from 870 to 994 °C (Table A3 Supplementary), and are generally in good agreement with mineral thermometry. For the four plagioclase-hosted melt inclusions that have retained their volatile contents, we have also calculated plagioclase-melt temperatures using the algorithm of Putirka (2005). These range from 839 to 972 °C, within 40 °C of the apatite saturation temperatures.

#### Volatile saturation pressures

We calculated H_2_O and CO_2_ saturation pressures for melt inclusions and interstitial glasses at the calculated apatite saturation temperatures (Table A3 Supplementary) and *f*O_2_ = NNO + 1 using the MagmaSat algorithm of Ghiorso and Gualda (2015). Values range from 0.5 to 3.8 kbar, with fluid compositions ranging from *X*H_2_O of 0–1. There is no correlation between calculated pressures and host mineral or xenolith textural type. However, it is striking that the interstitial glasses from KS-15 with high CO_2_ and low H_2_O record similar pressures (2.6 and 2.8 kbar) to the melt inclusions. This behaviour is suggestive of flushing of the magma with CO_2_-rich fluids prior to eruption (e.g. Blundy et al. 2010), displacing the interstitial melts along isobars, but without affecting the melt inclusions due to very slow intracrystalline diffusion of CO_2_.

#### Multiple reaction barometry

Two of the St. Kitts cumulate xenoliths (KS17, KS7) have the assemblage spinel–clinopyroxene–olivine–plagioclase, which has been developed as a geobarometer for mafic rocks using a multiple reaction method (Ziberna et al. 2017). We calculated pressures using mineral analyses from immediately adjacent, texturally equilibrated grains. The values are 6.4 ± 1.8 kbar for KS-7 and 6.0 ± 1.2 kbar for KS-17, lying at the upper limit of volatile saturation pressures from other xenoliths. Unfortunately, there are no melt inclusions in KS17, KS7 with which to make direct comparisons of these two methods.

### Experimental petrology

Experimental run conditions and phase proportions are given in Table 3, along with relative Na loss, and H_2_O and CO_2_ content of quenched glasses analysed by SIMS. For runs in which volatile contents of quenched glass could not be analysed because of an abundance of crystals and very small pool sizes, H_2_O and CO_2_ concentrations were estimated using MagmaSat (Ghiorso and Gualda 2015). In the four experiments where SIMS analyses were possible, the measured values and those calculated using MagmaSat are in good agreement (Table 3). All experiments are vapour-saturated as evidenced by presence of vapour bubbles in quenched run products. In three water-saturated runs (*a*H_2_O = 1.0), H_2_O concentration in the melt is around 6 wt% with 16–30 ppm CO_2_, likely introduced as a trace contaminant in the starting materials. The obtained data are in very good agreement with water solubility data of Botcharnikov et al. (2006) for andesitic melts at 200 MPa and 1100–1300 °C. The composition of the coexisting fluid was determined from the composition of the fluid added to the starting material, the measured glass volatile contents and the glass fraction determined by mass balance. As expected, the equilibrium fluid composition is H_2_O-poor compared to the starting material because of the greater solubility of H_2_O compared to CO_2_.

All experiments are crystallisation experiments where crystals nucleate and grow from the melt. No reversals were performed. Consequently, equilibrium in each individual experiment cannot be proven unequivocally. However, systematic variations in melt chemistry, melt fraction and mineral assemblages with changing temperature, together with homogeneous phase compositions, suggest close approach to equilibrium. Likewise, observed crystal textures and morphologies argue against significant problems with nucleation. Phases are homogeneously distributed throughout the capsule and crystals have euhedral shapes, although in two experiments (Run1#2 and Run2#2), skeletal orthopyroxene is suggestive of rapid growth. Residuals from mass balance calculations show that bulk silicate composition was maintained successfully with apparent Fe loss or gain less than 1%. Sodium loss is apparent in some crystal-rich runs with reduced *a*H_2_O. This is likely a result of focused-beam EPMA, although we cannot rule out some Na loss to the fluid, as inferred from Na systematics in natural St. Kitts glasses. No quench crystals occur in any of the run product glasses. Crystalline phases and glass were analysed by microprobe (Table 5) in all but two experiments Run3#3 and Run4#3 where the crystals and glass pools were ~ 1 micron. Amphibole in Run3#2 was too small to gain reliable analyses.

**Table 5:** Electron microprobe analyses of run products

Run no	Phase	*n*	SiO_2_	TiO_2_	Al_2_O_3_	Cr_2_O_3_	FeO*	MnO	MgO	CaO	Na_2_O	K_2_O	P_2_O_5_	NiO	Total	
Run1-1	melt	20	50.76 (17)	0.87 (02)	17.53 (17)	0.00	7.98 (20)	0.21 (01)	3.73 (05)	7.95 (17)	3.23 (13)	0.45 (03)	0.11 (04)	0.02 (02)	92.86	Mg# 45
Run1-2	melt	15	53.73 (21)	1.20 (03)	16.59 (25)	0	8.73 (21)	0.23 (01)	2.89 (11)	6.39 (15)	4.21 (20)	0.66 (04)	0.24 (04)	0	94.87	Mg# 38
	ol	5	36.55 (31)	0	0.06 (02)	0	29.96 (57)	0.59 (09)	31.28 (28)	0.22 (04)	0.02 (01)	0		–	98.80	Fo# 65
	opx	2	52.24 (47)	0.46 (11)	2.70 (06)	0	18.58 (38)	0.59	22.59 (47)	2.00 (21)	0	0.02 (01)	0	–	99.24	En79Wo5
	cpx	5	49.78 (60)	0.79 (10)	4.02 (90)	0	12.65 (90)	0.49 (13)	14.15 (1.66)	16.02 (1.88)	0.32 (08)	0	0.04 (02)	–	98.31	En49Wo38
	pl	4	53.63 (72)	0.14 (10)	27.76 (1.12)	0	1.15 (12)	0	0.31 (09)	11.36 (30)	4.0 (12)	0.13 (03)	0	–	98.53	An# 61
	spinel	5	0.42 (23)	11.98 (43)	4.90 (06)	0.26 (05)	72.45 (89)	0.44 (03)	3.18 (09)	0.32 (05)				–	94.02	Cr# 3
Run1-3	melt	11	57.50 (86)	1.61 (24)	14.81 (33)	0	9.20 (63)	0.39 (16)	1.92 (17)	5.64 (28)	1.88 (24)	1.34 (18)	0.28 (15)	–	94.26	Mg# 25
	cpx	5	49.97 (92)	0.69 (10)	2.22 (54)	0	16.77 (75)	0.65 (14)	15.12 (90)	13.24 (74)	0.19 (05)	0.02 (01)		–	98.91	En52Wo15
	pl	7	54.50 (78)	0.16 (09)	27.52 (1.13)	0	1.35 (45)	0	0.28 (17)	11.42 (28)	4.37 (26)	0.18 (03)	0.06 (04)	–	99.9	An# 58
	spinel	4	0.33 (04)	16.71 (17)	3.37 (02)	0.18 (03)	71.24 (44)	0.53 (12)	2.47 (02)	0.32 (04)					95.21	Cr# 3
Run2-1	melt	20	51.10 (31)	0.88 (02)	17.67 (13)	0	7.69 (13)	0.21 (02)	3.63 (11)	7.77 (10)	3.24 (08)	0.45 (02)	0.12 (03)	0	92.78	Mg# 46
	cpx	6	47.51 (72)	1.29 (29)	6.65 (60)	0.09 (02)	8.79 (18)	0.26 (01)	13.30 (46)	20.14 (25)	0.31 (04)	0	0	0	98.37	En44Wo48
	spinel	4	0.49 (20)	6.97 (17)	6.77 (12)	0.84 (32)	74.43 (33)	0.38 (09)	3.80 (10)	0.32 (16)	–	–	–	–	94.08	Cr# 8
Run2-2	melt	5	59.31 (1.45)	0.80 (18)	17.84 (80)	0	6.7 (71)	0	1.83 (27)	5.20 (39)	2.5 (64)	0.56 (03)	0.14 (08)	–	94.185	Mg# 23
	cpx	4	49.67 (26)	0.83 (11)	3.61 (47)	0	11.82 (70)	0.45 (14)	14.15 (13)	17.73 (60)	0.28 (02)	0	0	–	98.58	En47Wo43
	opx	5	51.20 (28)	0.42 (10)	2.51 (25)	0	19.32 (36)	0.63 (09)	21.85 (19)	2.20 (18)	0	0	0	–	98.19	En77Wo17
	pl	6	53.60 (1.11)	0.20 (15)	27.13 (1.63)	0	1.50 (72)	0	0.41 (26)	11.46 (98)	3.62 (57)	0.45 (03)	0.09 (06)	–	98.15	An# 62
	spinel	5	0.39 (08)	12.76 (24)	4.18 (06)	0.11 (02)	73.45 (56)	0.47 (09)	2.86 (02)	0.30 (06)					94.55	Cr# 2
Run2-3	melt	2	60.16 (92)	1.08 (24)	18.93 (31)	0	6.07 (18)	0	0.88 (06)	6.59 (22)	2.96 (16)	0.94 (05)	0	–	98.03	Mg# 21
	cpx	1	49.38	0.7	3.8	0	15.91	0.68	12.33	16.16	0.3	0.03	0.06	–	99.34	En44Wo41
	cpx1	8	50.77 (41)	0.66 (56)	1.83 (45)	0	22.57 (1.51)	0.82 (10)	15.52 (78)	6.75 (2.17)	0.14 (03)	0	0	–	99.09	En59Wo23
	pl	2	55.59 (09)	0.14 (03)	28.11 (07)	0	1.04 (09)	0	0.17 (09)	10.89 (51)	4.22 (48)	0.23 (03)	0	–	100.49	An# 58
	spinel	2	0.54 (21)	17.47 (10)	2.98 (08)	0.15 (05)	70.80 (67)	0.53 (05)	2.06 (08)	0.33 (03)				–	94.90	Cr# 3
Run3-1	melt	11	55.04 (31)	0.64 (03)	17.37 (40)	0	6.37 (16)	0.22 (01)	2.39 (11)	6.23 (25)	3.39 (19)	0.58 (03)	0.19 (03)	0	92.43	Mg# 40
	amph	6	43.26 (32)	2.16 (05)	12.70 (21)	0	11.77 (21)	0.25 (01)	14.04 (16)	10.57 (17)	2.48 (08)	0.18 (02)	0.01	0	97.42	Mg# 68
	cpx	1	53.76	0.58	8.92	0	8.50	0.38	11.24	15.10	0.63	0.23	0.08	0	99.44	En46Wo45
	pl	4	48.76 (42)	0.16 (04)	30.34 (76)	0	1.72 (23)	0.05 (01)	0.38 (10)	13.88 (12)	2.37 (20)	0.13 (02)	0.03 (02)	0	97.85	An# 76
	spinel	2	0.57 (18)	7.22 (15)	5.36 (06)	0.06	74.86 (10)	0.48	3.06 (11)	0.36 (07)				0.04 (02)	92.04	Cr# 0.7
Run3-2	melt	3	61.34 (2.21)	1.00 (30)	15.21 (32)	0	5.83 (71)	0.20 (07)	1.27 (28)	3.85 (36)	0.75 (42)	0.54 (24)	0.38 (03)	–	90.39	Mg# 28
	cpx	5	49.64 (1.59)	0.83 (12)	4.15 (1.39)	0	13.20 (1.16)	0.47 (05)	13.50 (1.78)	15.83 (1.29)	0.31 (12)	0	0	–	98.04	En48Wo12
	pl	5	53.87 (1.17)	0.10 (06)	28.75 (75)	0	0.78 (16)	0	0.10 (03)	11.54 (96)	4.43 (33)	0.13 (03)	0.02	–	99.76	An# 60
	spinel	6	0.44 (20)	13.76 (24)	3.61 (10)	0.10 (06)	73.73 (84)	0.54 (08)	2.34 (07)	0.26 (06)					94.84	Cr# 2
Run4-1	melt	6	56.56 (50)	0.55 (03)	17.13 (60)	0	5.69 (16)	0.22 (01)	1.83 (07)	5.76 (34)	3.49 (22)	0.69 (03)	0.20 (02)	0	92.17	Mg# 36
	amph	11	43.04 (74)	2.08 (13)	12.28 (88)	0	12.74	0.31 (01)	13.60 (71)	10.42 (21)	2.38 (08)	0.18 (02)	0.02 (01)	0	97.07	Mg# 66
	pl	3	49.11 (21)	0.05 (03)	31.36 (27)	0	0.99 (14)	0.02 (01)	0.17 (04)	14.15 (34)	2.75 (11)	0.07 (03)	0	0.02 (01)	98.72	An# 74
	spinel	5	0.61 (25)	7.78 (11)	4.53 (06)	0.03 (02)	76.03 (26)	0.52 (01)	2.46 (09)	0.39 (07)				0.01 (01)	92.4	Cr# 0.4
Run4-2	melt	7	60.45 (67)	0.76 (16)	16.15 (44)	0	5.33 (38)	0.17 (14)	1.25 (14)	4.23 (12)	1.19 (28)	0.67 (07)	0.23 (05)	–	90.46	Mg# 29
	amph	4	40.53 (48)	2.30 (32)	14.09 (65)	0	16.56 (97)	0.39 (16)	10.45 (28)	10.66 (14)	2.01 (03)	0.20 (03)	0.06 (04)	–	97.26	Mg# 53
	pl	10	53.49 (99)	0.09 (07)	28.76 (1.03)	0	1.01 (21)	0	0.16 (11)	11.94 (70)	3.90 (63)	0.11 (03)	0.06 (02)	–	99.55	An# 62
	spinel	8	0.36 (07)	13.25 (48)	3.58 (07)	0	75.07 (56)	0.54 (06)	2.32 (04)	0.28 (03)					95.66	Cr# 0

FeO* is iron total. Units in parentheses are standard deviation from average analyses, accordingly 8.88 (27) should be read as 8.88 ± 0.27

#### Phase relations

**Fig. 11:**
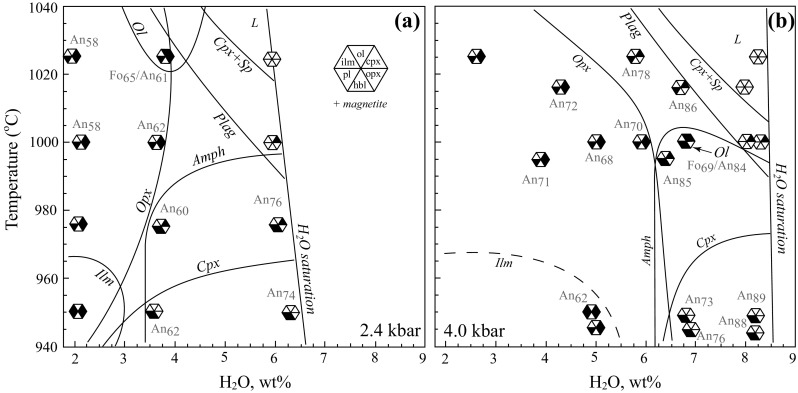
Phase diagrams for the experimental series at 2.4 kbar, this study (**a**) and 4 kbar, Pichavant et al. (2002a) (**b**). Hexagons show stable mineral assemblage: *ol* olivine, *cpx* clinopyroxene, *opx* orthopyroxene, *plag* plagioclase, *hbl* amphibole, *sp* spinel, *ilm* ilmenite, *L* liquid. Note expansion of orthopyroxene stability at 4 kbar compared to 2.4 kbar, and intersection (multiple saturation) of amphibole, orthopyroxene and plagioclase stability fields at 4 kbar

A phase diagram for experimental series with different *a*H_2_O is shown in Fig. 11a in terms of H_2_O in melt (H_2_O^melt^). Pichavant et al. (2002a, b) carried out experiments at 4 kbar on a basaltic andesite from Martinique (Table 2) that is very similar to the starting composition used in this study. Their experiments used a similar approach and can be usefully combined with ours (Fig. 11b).

The H_2_O-saturated liquidus for basaltic andesite is 1025 °C and 2.4 kbar (6 wt% H_2_O^melt^; Fig. 11a) and 1015 °C at 4 kbar (~ 8.5 wt% H_2_O^melt^; Fig. 11b). The 2.4 and 4 kbar phase diagrams have very similar topology. Their water-saturated crystallisation sequences are alike (olivine–clinopyroxene–plagioclase–amphibole), although the 4 kbar experiments lie closer to multiple saturation, with the latter three phases appearing within ~ 10 °C of the liquidus. Magnetite is present in all experiments and is a near-liquidus or liquidus phase. Clinopyroxene reacts out between 975 and 950 °C at both 2.4 and 4 kbar. In this region, amphibole is the only ferromagnesian mineral at elevated H_2_O^melt^.

The H_2_O-undersaturated liquidus is displaced to higher temperatures, but was not determined at either 2.4 or 4 kbar. With decreasing *a*H_2_O, plagioclase saturation is displaced to higher temperatures and amphibole to lower temperature. The limiting H_2_O^melt^ content for amphibole stability is 6 wt% at 4 kbar. At 2.4 kbar, the lower H_2_O^melt^ content for amphibole stability is less well defined, but likely lies just below 4 wt%. The field of clinopyroxene stability expands with decreasing H_2_O^melt^. A striking effect of reduced *a*H_2_O is the appearance of orthopyroxene for H_2_O^melt^ contents below 5 wt% at 2.4 kbar and 6.5 wt% at 4 kbar. The olivine stability field is very restricted, reflecting the relatively low MgO content of the starting composition. Olivine is present only at 1025 °C, 2.4 kbar and at 1000 °C, 4 kbar with H_2_O^melt^ of 3.8 and 6.8 wt%, respectively. Ilmenite is a low temperature phase, appearing below 960 °C at both pressures, but stable to higher H_2_O^melt^ at 4 kbar. The compositions of experimental run products show strong variation with intensive parameters (Table 5). The degree of crystallisation increases non-linearly with decreasing *a*H_2_O (Table 3).

#### Oxides

Titaniferous magnetite (TiO_2_ ~ 7–18%) is the dominant oxide phase forming euhedral crystals 1–10 μm in size in all experiments. In Run4#3, magnetite coexists with ilmenite. The TiO_2_ content of magnetite increases with decreasing H_2_O^melt^ (Table 5) and there is a slight negative correlation of TiO_2_ with temperature. Al# in magnetite increases and Fe^3+^# decreases with increasing temperature and H_2_O^melt^. Al# and Fe^3+^# concur with the data of Pichavant et al. (2002a, b) at comparable *f*O_2_ (Fig. 5). However, magnetite from this study is higher in TiO_2_ compared to magnetite from Pichavant et al. (2002a, b), probably because of slight differences in TiO_2_ content of starting compositions (Table 2). Magnetite in experiments HAB21, HAB20 and HAB23, HAB24 of Pichavant et al. (2002a, b) has a low-Al composition similar to that from KS-3 (Fig. 5) and distinct from the rest of the experimental magnetites. These four experiments were run under relatively oxidised conditions (ΔNNO + 3.1 to + 3.8) consistent with the textural inference that KS-3 experienced oxidation during differentiation, driving spinel towards magnesioferrite composition. Experimental magnetite compositions match natural phenocrysts.

*Olivine* (Fo_65_) is present in one experiment at 2.4 kbar. It is high in CaO and relatively high in MnO (Fig. 4). The 4 kbar olivine (run HAB7) of Pichavant et al. (2002a, b) is slightly more magnesian (Fo_69_) but a little lower in CaO and MnO. Experimental olivines match phenocrysts from St. Kitts lavas and cumulate xenoliths, but differ from plutonic xenoliths in terms of CaO and MnO contents (Fig. 4).

#### Pyroxenes

Clinopyroxene compositions range from diopside to augite. In Run2#3, diopside coexists with pigeonite. Clinopyroxene is high in Al_2_O_3_ (Al^IV^ from 0.08 to 0.21 apfu) and correlates positively with Mg# (Fig. 6b) and H_2_O^melt^, which probably reflects the delay in the onset of plagioclase crystallisation. Ca contents are strongly correlated with Mg# (Fig. 6a). Clinopyroxene in the 4 kbar experiments of Pichavant et al. (2002a, b) has higher Ca and Mg# than at 2.4 kbar (Fig. 6a). Orthopyroxene is enstatite with low Al_2_O_3_ (1.8–2.7 wt%). There is a decrease of Al^IV^ and Mg# and an increase of TiO_2_ with deceasing *a*H_2_O (Table 5, Fig. 6c). Ca increases with decreasing Mg# (Fig. 6d). In general, clinopyroxene that coexists with orthopyroxene has lower Ca contents, due to buffering along the pyroxene solvus. Coexisting pyroxenes from Runs #1–2 and 2–2 yield two-pyroxene temperatures, using Eq. (37) of Putirka (2008), of 1056 and 1028 °C, respectively. The Mg# range of experimental and natural pyroxenes is similar. Experimental clinopyroxene is displaced to lower Ca and higher Al^IV^ than its natural counterpart, whereas orthopyroxene is higher in Al^IV^ and Ca. The closest match to natural lava pyroxenes is found in H_2_O-saturated experiments at 4 kbar, 1000 and 1016 °C (HAB24 and HAB18, Pichavant et al. 2002a, b) and at 2.4 kbar, 1000 °C (Run2#1).

*Plagioclase* compositions range from An_58_ to An_74_ at 2.4 kbar (Fig. 1b) and from An_62_ to An_89_ at 4 kbar (Pichavant et al. 2002a, b). Plagioclase shows a negative correlation between K_2_O wt% and An (Fig. 7). Pichavant et al. (2002a, b) used a starting composition that had about 50% more K_2_O than KS_BR1 (Table 2), resulting in elevated plagioclase K_2_O content. Anorthite content increases with increasing H_2_O^melt^ at a given temperature, but does not change significantly with temperature at fixed H_2_O^melt^ (Fig. 11). For example, at 950 °C and 4 kb, An falls from 89 mol % at 8 wt% H_2_O^melt^ to 62 mol % at 6 wt% H_2_O^melt^; at 2.4 kbar the difference between runs with H_2_O^melt^ of 6.4 and 3.8 wt% is An_74_ and An_62_, respectively. Conversely, the difference between An content of plagioclase at 1025 and 950 °C in 2.4 kbar experiments with 3.8 wt% H_2_O is within analytical error. Experiments at 2.4 kbar failed to replicate the very high An content of natural plagioclases. The most calcic experimental plagioclase is An_89_ in an experiment at 4 kbar, 950 °C. According to the phase diagram in Fig. 11b plagioclase is stable to higher temperatures than 950 °C at 8.5 wt% H_2_O^melt^, so plausibly even more calcic plagioclase could form under these conditions. However, we consider it unlikely that plagioclase with more than 99 mol % An could ever crystallise from Na-bearing silicate melts, unless the topology of the plagioclase binary changes dramatically at An-rich compositions, as proposed by Nekvasil et al. (2015).

*Amphibole*, analysed in three run products, has 12.3–14.1 wt% Al_2_O_3_, Mg# of 52–68, 0.23–0.25 Ti apfu and 5.9–6.2 Si apfu. Mg# decreases with decreasing melt fraction. Amphibole in Run3#1 and Run4#1 is magnesiohornblende; in Run4#2 it is tschermakite (Leake et al. 1997). Amphibole–plagioclase temperatures (Holland and Blundy 1994) lie within 33 °C of experimental temperatures for all three runs. Experimental amphibole has compositions very similar to amphiboles from cumulate xenoliths KS-15 and KS-24, but distinct from plutonic amphiboles, notably in terms of Al^IV^ (Fig. 8 and Table 4 and A2 Supplementary), consistent with higher experimental temperatures than those calculated for plutonic xenoliths (Table 4).

*Melt* compositions change systematically from basaltic andesite through andesite to dacite with increasing crystallinity (Fig. 9). The effect of *a*H_2_O on plagioclase saturation imparts a strong influence on Al_2_O_3_ and CaO contents of experimental melts. At a given MgO content, Al_2_O_3_ and CaO in the melt increase linearly with H_2_O^melt^. Overall there is very close agreement between experimental melts and natural lavas and glasses with no discernible difference between 2.4 and 4 kbar experiments. There is some scatter in experimental TiO_2_ and FeO_tot_ contents both of which show some dependence on *a*H_2_O, reflecting changes in oxide phase composition and proportion. As lower *a*H_2_O corresponds to lower *f*O_2_ (Table 3), this makes melt composition strongly dependent on redox state. For example, the highly oxidised experiments (> NNO + 3) of Pichavant et al. (2002a, b) produced a magnetite-rich solid assemblage and correspondingly Fe-poor melt. The trend of decreasing Na_2_O at MgO contents < 3 wt% observed in lavas and natural glasses is reproduced in the experiments, although as noted above this may have its origins in Na loss from low melt fraction experimental glasses.

## Petrogenesis of St. Kitts xenoliths

Xenoliths, lavas and melt inclusions combined with experimental results provide constraints on magmatic processes and conditions beneath St. Kitts. Thus far, we have shown that:

1. The lavas, xenoliths, and experimental assemblages have a similar mineralogy, though lavas lack amphibole phenocrysts, orthopyroxene is more common in plutonic xenoliths and lavas than in cumulate xenoliths, and olivine is rare in experiments;
2. The prevalence of zoning in minerals in plutonic xenolith and lavas testify to a more complex magmatic history than the relatively unzoned cumulate xenolith minerals;
3. Some minerals (e.g. olivine, amphibole, oxides) are compositionally similar in experiments and specific sets of natural samples (i.e. lavas, cumulates or plutonics), whereas others (plagioclases, pyroxenes) are not;
4. Intensive parameters for xenoliths and lavas are very variable, as are estimates of fluid composition (*X*H_2_O);
5. Experimental melt compositions are broadly consistent with lavas regardless of P, T or *a*H_2_O.

In this section, we draw inferences from these similarities and differences in terms of magmagenesis on St. Kitts.

### Liquid lines of descent

The experimental liquids reproduce very well major element compositional variation of St. Kitts liquid line of descent (Fig. 9) as recorded by St. Kitts lavas (Toothill et al. 2007; Turner et al. 1996; and Baker 1984) and melt inclusions (Table A3 Supplementary and Toothill et al. 2007). The match between experimental liquids produced by water-rich experiments at 2.4 (this study) and 4 kbar (Pichavant et al. 2002a, b) allow us to speculate that St. Kitts lavas are consistent with being products of an oxidised (NNO to NNO + 1) parental basaltic andesite melt with high initial H_2_O content and relatively low CO_2_. This is especially evident from TiO_2_ and FeO_total_ variations as the higher TiO_2_ and FeO_total_ contents observed for water-undersaturated runs exceed those of St. Kitts lavas. This is largely a consequence of TiO_2_ content of melts being a function of *a*H_2_O (Melekhova et al. 2015), whereas FeO_total_ is a function of *f*O_2_ and controlled by magnetite saturation and composition.

Alkali contents are very sensitive to melt fraction and crystallising assemblages. Extensive crystallisation of pyroxene and delayed crystallisation of amphibole in runs with *a*H_2_O = 0.66 led to higher total alkalis. The trend of decreasing Na_2_O in natural samples with < 2.5 wt% MgO may be a consequence of partitioning of sodium into a coexisting vapour phase. Unfortunately, our experimental data, some of which are compromised by Na-loss during EMP analysis, alone do not allow us to quantify Na fluid-melt partition coefficients with any precision.

Toothill et al. (2007) demonstrated that melt inclusions in clinopyroxene, orthopyroxene, amphibole and plagioclase from lavas plot at higher SiO_2_ and total alkali abundances and lower Al_2_O_3_, CaO, TiO_2_ and FeO_total_ than the host lavas. Cumulate-hosted melt inclusions in orthopyroxene, plagioclase, amphibole and ilmenite replicate the melt inclusion lava trends. Overall, melt inclusions in phenocrysts from lavas and cumulates show compositions consistent with them being fractionated products of basaltic andesitic magmas similar to the experimental starting composition.

Toothill et al. (2007) and Macdonald et al. (2000) identified two distinct lava trends in St. Kitts, one characterised by higher Al_2_O_3_ and CaO and the other with lower Al_2_O_3_ and CaO (Fig. 9). The two trends diverge at approximately 4.5 wt% MgO, but both lineages extend to andesitic compositions (Fig. 9). At ~ 3 wt% MgO the high-Al group haa ~ 21 wt% Al_2_O_3_, while the low-Al group has ~ 18 wt% (Table 6 of Toothill et al. 2007). Macdonald et al. (2000) proposed that high-Al_2_O_3_ trend is the result of delayed plagioclase crystallisation, whereas enrichment in CaO is due to delayed clinopyroxene crystallisation. Conversely, Toothill et al. (2007) suggested that high-Al group derives from a “genuinely more aluminous [parent] magma type”.

Neither set of experiments was able to reproduce the highest Al and Ca basalts reported by Toothill et al. (2007). However, at a given MgO content our experimental melts show a striking correlation between Al_2_O_3_ and H_2_O^melt^ owing to the ability of water to suppress plagioclase saturation and hence prevent Al enrichment in derivative melts (Pichavant and Macdonald 2007, their Fig. 6). Taking only experimental melts with 3.0–4.2 wt% MgO (on an anhydrous basis) we find the following linear relationship (concentrations expressed at wt%):

2 $${\text{Al}}_{ 2} {\text{O}}_{ 3} \left( {{\text{anhyd}} .} \right) = 0.50(4) \, \times {\text{ H}}_{ 2} {\text{O}}^{\text{melt}} + 15.7(3)\left( {r^{2} = 0.834} \right).$$

**Fig. 12:**
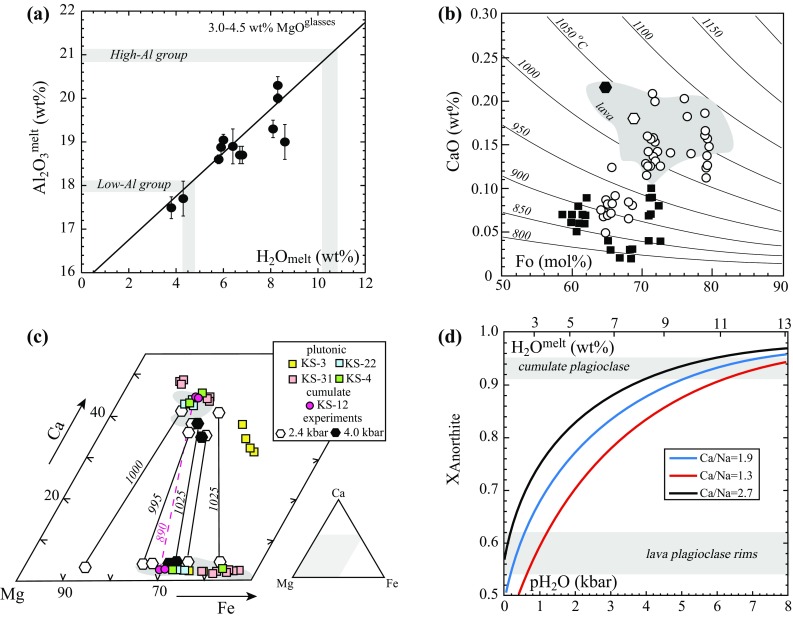
Independent constraints on magmatic temperatures and H_2_O contents. **a** Al_2_O_3_ contents of melts as a function of dissolved H_2_O from experiments presented in this study filtered for MgO contents of 3–4.5 wt%. Grey bars show Al_2_O_3_ contents of high-Al and low-Al groups of Toothill et al. (2007) at comparable MgO. **b** Temperatures of olivine-clinopyroxene equilibrium for experiments, xenoliths and lavas calculated using Ca-in-olivine thermometer of Shejwalkar and Coogan (2013). Symbols as in Fig. 4 with grey field to denote olivine + clinopyroxene-bearing lavas. Note the lower temperatures of plutonic xenoliths compared to lavas, experiments and most cumulate xenoliths. **c** Temperatures of coexisting orthopyroxene and clinopyroxene from experiments, lavas and xenoliths plotted in terms of Ca, Mg and Fe cations per formula unit. Tie-lines connect coexisting pyroxenes from experiments (labelled with experimental temperature). A single tie-line for a cumulate xenolith (KS-12) is shown. Note the lower temperature of lava and plutonic xenolith clinopyroxenes relative to experiments as shown by relatively high Ca. **d** Calculated An content of plagioclase as a function of H_2_O saturation pressure (pH_2_O) for three different molar Ca/Na ratios using the Kd_Ca–Na_ parameterisation in Eq. (3a). The upper axis indicates corresponding H_2_O^melt^ calculated for a representative basaltic andesite melt at 1000 °C using MagmaSat (Ghiorso and Gualda 2015). Grey bars denote plagioclase cores from cumulate xenoliths and phenocryst rims from lavas

Toothill et al’s (2007) high-Al group would require H_2_O^melt^ of 10.6 ± 1.0 wt% and their low-Al group 4.6 ± 0.4 wt% (Fig. 12a). Thus, the primary control on the different trends observed by Toothill et al. (2007) can be ascribed simply to differences in magmatic H_2_O contents obviating the need to invoke more (and less) aluminous parent magma types. The two groups may originate from the same H_2_O-rich parental magma differentiated under water-saturated conditions at different crustal depths (pressures). For a typical St. Kitts basalt with 3.5 wt% MgO, 10.6 wt% H_2_O^melt^ would correspond to saturation at 5.8 kbar and 4.6 wt% H_2_O^melt^ to 1.6 kbar, based on calculations using MagmaSat (Ghiorso and Gualda 2015). On this basis, we propose that the different Al_2_O_3_ trends observed at St. Kitts represent different differentiation pressures of one or more water-rich basaltic andesite magmas that are themselves products of differentiation of more magnesian basalts generated in the mantle wedge. The proposed pressure range is consistent with that determined from cumulate (1.0–6.4 kbar) and melt inclusion (0.5–3.9 kbar) barometry. Our failure to reproduce the high-Al lava trend experimentally then stems simply from a failure to perform water-saturated experiments at sufficiently high pressure.

### Xenolith whole-rock chemistry

We show also in Fig. 9 whole-rock major element compositions of plutonic and cumulate xenoliths calculated from point-counted mineral modes and mineral compositions, as well as the experimental solid residues recalculated from phase proportions and mineral compositions. As expected, experimental residues lie on the low-SiO_2_ extrapolation of tie-lines linking experimental melts to the relevant starting composition. In contrast, neither plutonic nor cumulate xenolith compositions overlap the experimental solids or any natural rock compositions from St. Kitts, indicating that the xenoliths are not simply crystal extracts driving the observed liquid lines of descent. Chemically the xenoliths cluster into two groups in keeping with the textural criteria used to subdivide them.

Cumulate xenoliths are significantly richer in Al_2_O_3_ and CaO and poorer in Na_2_O and SiO_2_ than experimental residues (Fig. 9). This discrepancy, which is surprising in light of the close match of the experimental liquids to St. Kitts lavas, arises because the experimental assemblages consistently have higher proportions of clinopyroxene, at the expense of amphibole, than the cumulates (Fig. 1). We consider three possible explanations for the discrepancy: in the first the cumulates are products of crystallisation of a parent magma less evolved than the basaltic andesite experimental starting material; in the second differentiation took place at pressures higher than the experiments (see above); finally, cumulate compositions have been modified by cryptic chemical interaction with migrating melts and/or fluids in the magmatic system, which converted early formed clinopyroxene into amphibole. All three explanations have merit, and will be explored more fully below.

The plutonic xenoliths also plot outside the range defined by whole rock data of St. Kitts lavas, having lower SiO_2_ and total alkalis, and higher Al_2_O_3_ and CaO (Fig. 9). Consequently, the plutonic xenoliths cannot be simply solidified versions of erupted lavas. Nonetheless, the complex mineral zoning and reaction textures and diverse mineral assemblages of the plutonic xenoliths are suggestive of protracted crystallisation of magma and reaction with trapped melt. Interestingly, the bulk compositions of the plutonic xenoliths lie intermediate between the cumulate xenoliths and the lavas themselves. Thus, a simple explanation for the plutonic xenoliths is that they represent mixtures of cumulates (irrespective of their origin) with varying proportions of trapped melt of the type represented by the diverse erupted melts. The plutonic xenoliths are, in effect, fragments of magmatic mush, in which migrant melts have become trapped, driving a plethora of reactions. Protracted cooling and crystallisation of these mushes within the sub-volcanic reservoir can account for both their complex textures and their relatively low crystallisation temperatures.

### Mineral compositions

There are significant mismatches between experimental mineral assemblages and compositions, lavas and xenoliths. In this section, we explore possible explanations for these differences using compositions of phenocrysts from St. Kitts lavas (Toothill et al. 2007; Turner et al. 1996; Baker 1984), minerals from xenoliths, and experimental run products.

*Olivine* phenocrysts in lavas are in the range of Fo_83–63_ with CaO content > 0.13 wt%, whereas xenolith olivines lie in the range Fo_80–58_, but with much more variable CaO (0.02–0.21 wt%; Fig. 1a). The lowest CaO content olivines are found in plutonic xenoliths. Experimental olivines lie within the field defined by phenocrysts and most cumulate xenoliths, although their Fo contents (65–70 mol % Fo) are at the lower end of the natural range. Evidently some xenolith and lava olivines crystallised from magmas slightly more magnesian than our basaltic andesite starting composition.

Olivine CaO depends on a variety of magmatic parameters, e.g. melt composition, temperature, pressure (e.g. Jurewicz and Watson 1988; Köhler and Brey 1990; Kamenetsky et al. 2006; Mysen 2004). However, in the presence of clinopyroxene, the CaO content of olivine is controlled primarily by temperature (Köhler and Brey 1990; Shejwalkar and Coogan 2013). In Fig. 12b we have contoured the CaO versus Fo plot (Fig. 4a) for temperature using the thermometer Eq. (12) of Shejwalkar and Coogan (2013). The high CaO contents of olivine in experiments, lavas, and some cumulate xenoliths are consistent with crystallisation from relatively high-temperature melts (1025–1125 °C). Thus, phenocrystic olivines appear to be high-temperature primocrysts crystallised at or close to the liquidus. Conversely, all plutonic xenoliths and some cumulates have lower CaO contents consistent with re-equilibration with clinopyroxene down to temperatures as low as 800 °C. This supports our contention that plutonic xenoliths (and some cumulates) have undergone cooling and solidification in the sub-volcanic reservoir. In the case of KS-3 olivine oxidation, to form orthopyroxene-oxide symplectites, may have increased olivine Fo content slightly (Johnston and Stout 1984). Oxidative increase in Fo can also account for the displacement of these olivines on the MnO–Fo plot (Fig. 4b). Thus, we suggest that the distinctive behaviour of plutonic olivine is a consequence of partial modification of their Fo and CaO contents during both cooling and oxidation.

#### Oxides

There is close compositional correspondence between experimental spinels and those in cumulates, although the latter extend to much higher Al# and lower Fe^2+^/(Mg + Fe^2+^) (Fig. 5). The higher Al# of cumulate spinels likely reflects the higher Al content of the coexisting melts, which we attribute above to elevated magmatic H_2_O contents suppressing plagioclase crystallisation relative to olivine and clinopyroxene. Thus, cumulate spinel testifies to crystallisation from slightly wetter and more primitive magmas than the basaltic andesite starting material. Spinel phenocrysts in lavas overlap with low Al# end of the experimental and cumulate spinel, and provide a good match with spinel in plutonic xenoliths that lack evidence for oxidation.

**Fig. 13:**
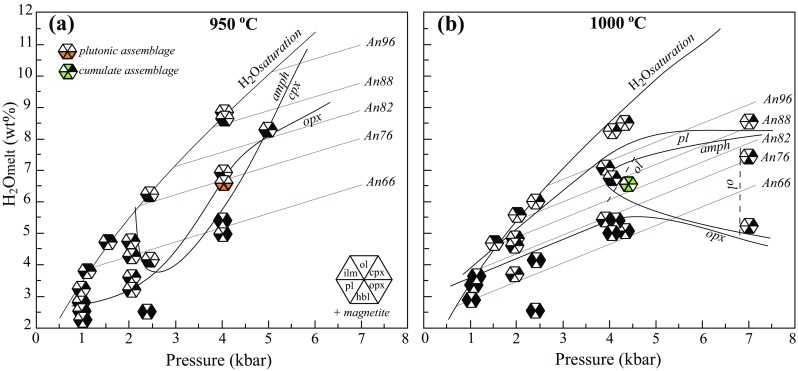
Phase diagrams at 950 and 1000 °C plotted as functions of pressure and H_2_O^melt^ for experiments from this study and published data from Pichavant et al. (2002a), Almeev et al. (2013), Erdmann et al. (2016), Grove et al. (1997) and Laumonier et al. (2017). Grey solid lines with An content of plagioclase are based on weighted least-squares regressions of all plotted plagioclase-bearing experiments. H_2_O saturation at run conditions was calculated with MagmaSat (Ghiorso and Gualda 2015). Assemblages shown in orange and green refer to typical plutonic and cumulate xenolith assemblages, respectively, from St Kitts. Note effect of temperature on olivine and orthopyroxene stability and universal stability of clinopyroxene at 1000 °C. Mineral abbreviations as in Fig. 11

*Pyroxenes* in cumulate and plutonic xenoliths and lavas show almost complete overlap in terms of Mg# for clinopyroxene (Fig. 6a) and orthopyroxene (Fig. 6c). Experimental clinopyroxenes intersect the xenolith array at high Mg#, but define differentiation trends to lower Ca and higher Al^IV^. Experimental orthopyroxene is displaced to slightly higher Ca and Al^IV^ contents. The Ca content of coexisting pyroxenes is buffered by the pyroxene solvus, which forms the basis of two-pyroxene thermometry (e.g. Wood and Banno 1973; Wells 1977; Lindsley 1983). Lower Ca in clinopyroxene and higher Ca in orthopyroxene reflect higher equilibration temperatures. Tie lines linking selected experimental and xenolith pyroxene pairs (Fig. 12c, Table 4) are consistent with Kd_Fe-Mg_ between orthopyroxene and clinopyroxene being slightly greater than one (Putirka 2008). Figure 13c indicates that the experimental pyroxene pairs are consistent with higher crystallisation temperatures (1000–1025 °C) than cumulates (− 900 °C), plutonics or lavas. The latter show very scattered compositions, even within a single sample (Toothill et al. 2007), that overlap with those of plutonic xenoliths, whose two-pyroxene crystallisation temperatures are in the range 770–940 °C (Table 4). The Ca-rich nature of phenocrystic clinopyroxene is therefore consistent with them being xenocrysts appropriated from cooler pockets within the magmatic mush system. A few lava clinopyroxene phenocrysts extend to lower Ca, suggestive of high-temperature crystallisation from the host magma. However, in contrast to olivine, the majority of phenocrysts record temperatures too low to reflect an origin as true primocrysts. The higher Al^IV^ contents of experimental pyroxenes (Fig. 6b, d) may also reflect higher crystallisation temperatures.

#### Amphibole

St. Kitts lavas are almost all amphibole-free, a common feature of Lesser Antilles islands with the exception of Grenada, Guadeloupe, and Saba (e.g. Arculus 1976; Westercamp and Mervoyer 1976; Baker 1980). Amphiboles in plutonic and cumulate xenoliths are texturally similar, but compositionally distinct, notably in Mg# and Al^IV^, which are proxies for melt composition (and/or *f*O_2_) and crystallisation temperature, respectively (Blundy and Holland 1990). Plutonic amphiboles have lower Mg# and Al^IV^ (Fig. 8a, b) than their cumulate counterparts, consistent with crystallisation to lower temperatures. Experimental amphiboles overlap the low Mg# and Al^IV^ of the cumulate amphibole array. Based on textural evidence, we propose that cumulate xenolith amphiboles (± calcic plagioclase) originate by percolative reaction between H_2_O-rich melts or fluids and anhydrous, pyroxene-dominated residues, similar to those in the experiments. The hydrous fluid may also lead to the observed olivine iddingsitisation in some cumulate xenoliths. A similar metasomatic origin for cumulate amphibole was advanced by Smith (2014) for Solomon Islands xenoliths, although their lack of plagioclase likely reflects higher temperatures and/or H_2_O contents.

#### Plagioclase

Lavas and xenoliths contain highly calcic plagioclase, An_95–54_ and An_97–72_, respectively. The plutonic xenolith xenoliths span an even wider range, An_100_–_37_, consistent with protracted cooling and crystallisation. Experimental plagioclase does not exceed An_72_ at 2.4 kbar, and An_89_ at 4 kbar. Reaching even higher An contents likely reflects crystallisation from hotter or wetter magmas and/or higher Ca/Na magmas. For example, Sisson and Grove (1993a, b) produce An_93_ plagioclase in 2 kbar, water-saturated crystallisation experiments on a high-Al basalt with molar Ca/Na = 2.9 and ~ 10 wt% MgO. However, these experiments are too hot (1050 °C) for amphibole saturation and co-crystallise instead Mg-rich olivine (Fo_84_) and clinopyroxene. The challenge for St. Kitts, therefore, is how to produce An-rich plagioclases from melts that are basaltic andesites with molar Ca/Na ratio (≤ 2.7) in keeping with erupted lava compositions (Turner et al. 1996; Toothill et al. 2007). In all St. Kitts cumulate xenoliths An_>90_ plagioclase co-crystallised with amphibole, olivine (Fo_<80_) and magnetite. Significantly, in some cumulates An_>90_ plagioclase also co-crystallised with orthopyroxene (Fig. 1), a rare association in other Lesser Antilles xenoliths (Arculus and Wills 1980). The assemblage plagioclase (An_97–85_) + orthopyroxene + clinopyroxene + olivine (Fo_65–70_) + magnetite + amphibole (e.g. KS-12; Fig. 2d) is unusual for experiments performed on basaltic andesite bulk compositions (e.g. Table 3, Sisson and Grove 1993a, b; Almeev et al. 2013; Erdmann et al. 2016).

We can explore possible mechanisms for generating An-rich plagioclase by considering the exchange of Ca and Na between plagioclase and melt from the experiments. For our new experiments and those of Pichavant et al. (2002a, b) we find the following exponential relationship between Kd_Ca–Na_ (= [Ca/Na]_plag_/[Ca/Na]_melt_) and H_2_O^melt^ (Fig. A1 Supplementary):

3a $${\text{Kd}}_{{{\text{Ca}} - {\text{Na}}}} = 0. 4 8 5 \pm 0.0 5 1 { }*{ \exp }\left\{ {0. 2 9 4 \pm 0.0 1 9 { }*{\text{ H}}_{ 2} {\text{O}}^{\text{melt}} } \right\}\left( {r^{ 2} = 0. 7 3 3} \right)$$

There is no significant temperature effect over the range of experimental temperatures studied. The slope of this relationship is similar to that reported by Sisson and Grove (1993a, b), although their data are offset to slightly higher Kd_Ca–Na_ than ours, due to differences in bulk composition. We emphasise that Kd_Ca–Na_ is very sensitive to melt composition (e.g. Sisson et al. 2005; Hamada and Fujii 2007), so Eq. (3a) should only be used for basaltic andesites similar to our experimental starting materials.

In Fig. 13d, we show the calculated variation in plagioclase An content for three different melt Ca/Na contents: the two starting materials (Table 2) and the maximum molar Ca/Na (= 2.7) of any St. Kitts lava (Kit59) as reported by Toothill et al. (2007). Kit59 is a Black Rocks basalt with Mg# = 59 and therefore in equilibrium with Fo_82_ olivine, the most Mg-rich observed in any St. Kitts natural sample (Fig. 4). The calculated variation in An with H_2_O^melt^ indicates that the highly calcic plagioclase of St. Kitts lavas and xenoliths requires magmatic H_2_O contents of 9–13 wt% (5–8 kbar saturation pressure), in good agreement with the value estimated independently from lava Al_2_O_3_ contents. The lower An rims of lava phenocrysts would require H_2_O^melt^ of < 1 wt%, consistent with them being primocrysts grown from their host magma during magma ascent and degassing.

Although high H_2_O melts may be responsible for very calcic plagioclase, it is worth mentioning other possibilities. For example, the An content of plagioclase can also be influenced by exchange of Ca and Na with a fluid phase, similar to that invoked for metasomatic amphibole crystallisation. Thus, the most calcic plagioclases in St. Kitts xenoliths may have a partially metasomatic origin that reflects dissolution and reprecipitation of plagioclase in the presence of migrating Na-poor melts or fluids. Conversely, Devine and Sigurdsson (1995) suggest that very calcic plagioclase (An_>95_) in the Lesser Antilles is the result of crustal assimilation, although Sr isotopic data (Toothill et al. 2007) argue against this on St. Kitts.

### Phase assemblages

In keeping with other Lesser Antilles islands (Arculus and Wills 1980) the diagnostic St. Kitts xenolith assemblage is calcic plagioclase + amphibole + clinopyroxene + spinel ± orthopyroxene ± olivine (Fig. 1a). A particular petrogenetic challenge is identifying a stability field for calcic plagioclase + orthopyroxene + amphibole ± olivine, an assemblage that was not generated under any of the experimental conditions investigated (Fig. 11). As cumulates are snapshots of the overall differentiation process, albeit modified by metasomatic reaction, it seems likely that some part of the P–T–H_2_O evolution was not captured experimentally. To explore possible conditions under which the St. Kitts xenolith assemblages were formed, in Fig. 13 we have compiled all published experimental data on bulk compositions similar to those of St. Kitts basaltic andesite (Grove et al. 1997; Pichavant et al. 2002a, b; Almeev et al. 2013; Erdmann et al. 2016; Laumonier et al. 2017; this study) at temperatures of 950 and 1000 °C (Fig. 13). The experimental starting compositions have Mg# 41–54 and molar Ca/Na from 2.3 to 3.8. The water saturation curve in Fig. 13 was calculated using MagmaSat (Ghiorso and Gualda 2015) for a representative basaltic andesite at the experimental temperature. At each experimental temperature we regressed plagioclase An content as a function of P and H_2_O^melt^ and contoured the plots accordingly.

Amphibole + plagioclase + clinopyroxene coexist over a wide P–H_2_O^melt^ range (Fig. 13). Clinopyroxene is stabilised at higher temperatures; with decreasing temperature clinopyroxene and amphibole are in a reaction relationship which is terminal to clinopyroxene crystallisation at pressures of 2–5 kbar. Olivine stability is not clearly defined at either 950 or 1000 °C, although we suspect that all melts are close to olivine saturation and that for slightly higher MgO starting materials the olivine field would expand significantly. Orthopyroxene stability is diminished at high *f*O_2_ and high H_2_O^melt^ (Sisson and Grove 1993b). At 1000 °C orthopyroxene is only stable when H_2_O ≤ 5wt % at 4 kbar (Fig. 13b). At 950 °C the upper pressure limit of orthopyroxene stability is unconstrained, but certainly exceeds H_2_O^melt^ = 8 wt% and 5 kbar (Fig. 13a). The effect of H_2_O^melt^ on plagioclase saturation is evident at 1000 °C, where plagioclase does not crystallise from melts with more than 8 wt% H_2_O, whereas at 950 °C plagioclase can crystallise from melts with almost 9 wt% H_2_O. The typical plutonic and cumulate xenolith assemblages are shown with coloured symbols. In both cases they lie at P ≥ 4 kbar. However, experimental plagioclase compositions match only the more sodic rims in the natural rocks rather than the calcic cores (Fig. 1).

Although the mineral assemblages can be reproduced at 4 kbar, 950–1000 °C and H_2_O^melt^ ≈ 10 wt% the experimental plagioclase remains less calcic (An_82–76_) than observed in the cumulates (An_92–96_). As noted above, this reflects both the Ca/Na ratio of the experimental starting materials, and the tendency of plagioclase An content to increase with increasing P, T and H_2_O (Fig. 13). Anorthite content increases more rapidly with increasing H_2_O at 1000 °C compared to 950 °C. The highest An contents occur at high pressures and high H_2_O^melt^: An_92–96_ plagioclase is stable for H_2_O^melt^ in excess of 10 wt% at 950 °C and 6.5 wt% at 1000 °C. Co-precipitation of these calcic plagioclases with orthopyroxene, as observed in many St. Kitts xenoliths, is problematic, based on the available experimental data. The closest approach is at 950 °C at P > 5.5 kbar and H_2_O^melt^ ≥ 10 wt%. Crystallisation of An-rich plagioclase is enhanced from melts with high molar Ca/Na, although the maximum value on St. Kitts, based on lavas is 2.7. Lava compositions need not necessarily represent true liquids, especially when crystals are entrained, yet this process will only serve to artificially elevate Ca/Na ratios. Instead we propose, as inferred above based on plagioclase composition and lava Al_2_O_3_ content, that xenolith assemblages on St. Kitts require crystallisation from very H_2_O-rich basaltic or basaltic andesite magmas.

**Fig. 14:**
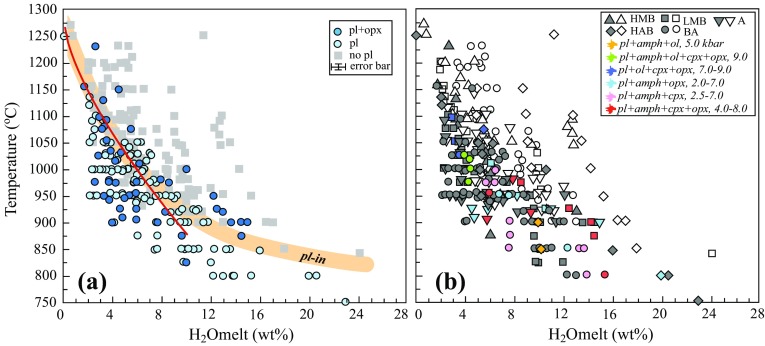
Compilation of experimental data showing plagioclase stability as a function of H_2_O^melt^ and temperature for pressures between 0.5 and 12 kbar in the following bulk compositions: high magnesium basalt (HMB), low magnesium basalt (LMB), high alumina basalt (HAB), basaltic andesite (BA) and andesite (A). When H_2_O concentration is not reported MagmaSat was used to estimate H_2_O^melt^ at the run conditions. A representative 1 s.d. uncertainty is shown. **a** Plagioclase and orthopyroxene stability field. The plagioclase-in (*pl*-*in*) line demonstrates extent of plagioclase stability in melts with H_2_O contents up to 24 wt%. **b** Experimental data shown in terms of the starting compositions (symbols) and run product assemblages and corresponding pressure in kbar (colours). Filled symbols denote the presence of plagioclase; open symbols are plagioclase-free. Some typical cumulate and plutonic xenolith assemblages from St Kitts (Table 1) are illustrated by different colours: orange—KS8, KS21, KS15; dark blue—KS12; pink—KS24; red—KS4; light blue—KS6. Solid orange line in (**a**) is plagioclase-in boundary of Almeev et al. (2012)

To explore whether such wet magmas can crystallise plagioclase we have compiled existing experimental data on a broad range of hydrous arc magma compositions at pressures ranging from 1 to 12 kbar (Fig. 14, Fig. A2 Supplimentary) (Almeev et al. 2013; Alonso-Perez et al. 2009; Beard and Lofgren 1990; Blatter and Carmichael 2001; Blatter et al. 2013; Brooker unpublished; Cawthorn et al. 1973; Eggler and Burnham 1973; Erdmann et al. 2016; Green and Ringwood 1968; Grove et al. 1997, 2003; Holloway and Burnham 1972; Laumonier et al. 2017; Melekhova et al. 2015, Müntener et al. 2001; Nandedkar et al. 2014; Panjasawatwong et al. 1995; Pichavant et al. 2002a, b; Prouteau et al. 2001; Rapp and Watson 1995; Sisson and Grove 1993a, b; Sisson et al. 2005; Winther 1990 (thesis); Yoder and Tilley 1962). The experimental starting compositions include high magnesium basalts (HMB), low magnesium basalt (LMB), high alumina basalt (HAB), basaltic andesite (BA) and andesite (A). In Fig. 14a we subdivide plagioclase-free melts from melts saturated with plagioclase or plagioclase + orthopyroxene. Plagioclase is stable in melts with as much as 20 wt% H_2_O, provided temperatures are low. Our subdivision agrees well with the formulation of Almeev et al. (2012) shown as an orange line on Fig. 14a. At 950 °C the upper H_2_O content of plagioclase-saturated melts is 9 wt%, consistent with inferences from Fig. 13. Orthopyroxene coexists with plagioclase under these conditions.

In Fig. 14b, we have subdivided the experiments on the basis of their starting materials. Plagioclase stability is clearly independent of magma composition for the different magma types considered. Assemblages identical to those of St. Kitts xenoliths form at pressures of 2–9 kbar. Basaltic andesites with 8–10 wt% H_2_O crystallise an assemblage of amphibole + plagioclase + orthopyroxene or amphibole + plagioclase + clinopyroxene at temperatures of 850–950 °C over a wide range of crustal pressures. Hydrous high alumina basalts under the same conditions crystallise the assemblage amphibole + plagioclase + olivine. Thus, the mineral assemblages of St. Kitts xenoliths are broadly consistent with crystallisation from H_2_O-rich basalts and basaltic andesites at pressures in excess of 4 kbar.

## Implication for arc magmatism

Petrological, mineralogical and experimental data provide four independent lines of evidence for differentiation of water-rich (5–11 wt% H_2_O) basaltic andesite magmas at mid-crustal depths beneath St. Kitts: highly calcic plagioclase; the mineral assemblage of cumulate xenoliths; mineral barometry; and the high-alumina group of magmas. In this section we interpret these findings in the context of a vertically extensive (“transcrustal”) magmatic mush within which hydrous, basaltic magmas supplied from the underlying mantle wedge crystallise and degas, and residual melts reactively percolate upwards (Cashman et al. 2017).

The elevated magmatic water contents are consistent with findings at a number of other arc volcanoes. Edmonds et al. (2016) propose 6–9 wt% H_2_O in andesites from Soufrière Hills Volcano, Montserrat, on the basis of H_2_O dissolved in orthopyroxene phenocrysts. Grove et al. (2003) proposed ≥ 11 wt% H_2_O in some primitive magmas from Mt. Shasta, Cascades, on the basis of amphibole chemistry and melt inclusions. Laumonnier et al. (2017) used a combination of geophysical and petrological evidence to argue that the andesitic partial melt contained within the Altiplano-Puna Magma Body contains 9–11 wt% H_2_O.

Our proposal for elevated H_2_O contents in St. Kitts basaltic andesites requires proportionately higher H_2_O in parental basalts. Experimental data (e.g. Nandedkar et al. 2014; Melekhova et al. 2015) show that basaltic andesites can be generated from mantle-derived MgO-rich basalts by approximately 40–60 wt% crystallisation, predominantly of anhydrous mafic silicates. In that case basaltic andesite H_2_O contents of ≤ 11 wt% would equate to ≤ 6.6 wt% H_2_O in the parental basalt. This value is within the range (≤ 7.2 wt%) of H_2_O contents of olivine-hosted basaltic melt inclusions from a broad selection of subduction zones (Plank et al 2013), for which the mean H_2_O content is 3.9 ± 0.5 wt%. Basaltic andesite H_2_O contents of > 7 wt% requires that differentiation of their parent magmas occurs at sufficient depth in the crust to hold H_2_O in solution. In the case of St. Kitts basaltic andesites ≤ 11 wt% H_2_O corresponds to pressures of ≤ 6.5 kbar (at 1050 °C). For a mean crustal density of 2660 kg/m^3^ for the Lesser Antilles arc (Christeson et al. 2008) this equates to lower crustal depths of ≤ 25 km.

The basaltic andesite starting materials from our experiments have been shown to provide a good match to parental melts for some St. Kitts xenoliths, although slightly higher Mg# parents are required to generate the observed olivine Fo contents. However, adding a small amount of olivine to the basaltic andesites will not render them sufficiently magnesian to be in equilibrium with mantle peridotite. Mantle-derived magnesian basalts would need to crystallise 40–60 wt% to generate the low-magnesium basalts or basaltic andesites found on St. Kitts. The crystalline residues from this differentiation would be olivine + clinopyroxene + spinel (Müntener et al. 2001; Müntener and Ulmer 2006; Nandedkar et al. 2014; Melekhova et al. 2015) with little or no plagioclase or amphibole. We suggest that cumulate rocks with this mineralogy underlie the St. Kitts magmatic system, at depths greater than 25 km. It is worth noting that the density of seismic velocities of such ultramafic cumulate rocks would make them very hard to distinguish geophysically from mantle rocks (Müntener and Ulmer 2006). It is perhaps for this reason that the Moho discontinuity is not clearly resolved beneath the Lesser Antilles (Christeson et al. 2008; Kopp et al. 2011).

We propose that water-rich, relatively oxidised (NNO + 1 to NNO + 2) low-Mg basalts and basalt andesites generated by olivine + clinopyroxene + spinel fractionation in the deep crust ascend to 18–25 km where they begin to differentiate, producing the xenolith varieties observed on St. Kitts. Elevated H_2_O contents are required to generate the calcic plagioclase that is diagnostic of xenoliths on St. Kitts and elsewhere in the Lesser Antilles. St. Kitts xenoliths record snapshots of this polybaric differentiation process, whereas erupted magmas represent the integrated products, modified by migration through the thick crystal mush pile. Our experiments show that most of the lavas erupted on St. Kitts can be generated at pressures of 2.4–4 kbar, with H_2_O contents down to 2–6 wt%, i.e. significantly lower than those inferred for the basaltic andesite parents. The tendency for erupted magmas to match low-pressure experimental data is consistent with the vertically extensive mush concept; as melts migrate upwards they will continually equilibrate with crystals in the mush. Their apparent equilibration pressure will correspond to the top of the mush, even though differentiation began at much greater depths. Only occasionally do melts bypass the mush column and so preserve their high-pressure chemistry upon eruption; the Al-rich series on St. Kitts is one such example.

We propose that the hallmark of magmas generated in transcrustal mushes will be multiple saturation with four or more of the following mineral phases: olivine, clinopyroxene, orthopyroxene, amphibole, plagioclase, and spinel. Conversely, the crystals represent snapshots of the entire, polybaric differentiation process and are, therefore, much more sensitive to local conditions. As basaltic andesite or low-Mg basalts magmas entering the base of the mush are H_2_O (and possibly CO_2_) rich, so the melts leaving the top of the mush will be volatile-saturated. Once a significant mass of mush is established in the arc crust, it tends to buffer melt compositions, becoming relative immune to new additions of slightly different composition. We suggest that the ability of the mush to buffer melts along polybaric, multi-phase cotectics gives rise to relatively well-defined liquid lines of descent. Recent experimental studies of a Mount St Helens dacite by Blatter et al. (2017) are a case in point; they find multiple saturation with amphibole–plagioclase–orthopyroxene–clinopyroxene–oxide at 4 kbar. The abundance of amphibole within the crystal mush column drives amphibole saturation with attendant trace element signatures, despite the paucity of amphibole phenocrysts in many erupted arc magmas (Davidson et al 2007). There is abundant textural evidence in St. Kitts xenoliths for percolative, reactive flow of hydrous fluids/melts, including reactions of early-formed clinopyroxene to amphibole, mineral zoning, poikilitic crystals and disequilibrium textures. The consequences of percolative reactive flow may be especially complicated for trace elements, as observed in xenoliths from Martinique (Cooper et al. 2017).

As melts ascend through transcrustal mush they will exsolve volatiles and interact with pre-existing crystals. Interactions include metasomatic reactions, such as those observed in plutonic xenoliths from St. Kitts. Ascending melts may also rip up mush fragments, either as crystal clots or as disaggregated xenocrysts. We have demonstrated that calcic plagioclase, spinel and pyroxene phenocrysts in St. Kitts lavas are typically xenocrystic (or more accurately, antecrystic) in origin. This crystal cargo may be significantly overgrown or overprinted by liquidus phases that crystallise from the host magma as it leaves the top of the mush and ascends to the surface. Olivine phenocrysts and intermediate plagioclase rims in St. Kitts lavas are examples of low-pressure primocrysts. In arc magmas, unraveling the relative proportions of xenocrysts, antecrysts and primocrysts is notoriously difficult.

The release of exsolving fluids from ascending magmas within the mush may also lead to reactions with pre-existing crystals, including dissolution and reprecipitation, leaching of fluid-mobile elements (such as Na) and production of hydrous mineral phases (e.g. Smith 2014). Because of their different densities, it is likely that fluid and melt movement are decoupled (Christopher et al. 2015). The compositions of the exsolved fluid changes with depth, being more CO_2_ (and SO_2_) rich at higher pressures. Percolation of fluids from the top of the mush that have been released across a considerable vertical depth range may explain the apparent flushing of shallow-stored magmas with CO_2_. There is widespread evidence of this in melt inclusions from arc volcanoes (e.g. Blundy et al. 2010) as well as in some interstitial glasses from St. Kitts xenoliths. Discharge of SO_2_-rich fluids from the top of a transcrustal mush may play an important role in hydrothermal mineralization (Mavrogenes and Blundy 2017).

The process of melt migration through the transcrustal magmatic mush can operate on two different timescales. Slow, percolative flow can account for many of the observed xenolith textures and the tendency for erupted magmas to be chemically buffered. However, the presence of xenoliths containing volatile-rich melt inclusions indicates that a more rapid process may also operate, whereby melts disaggregate and entrain mush shortly before eruption. Christopher et al. (2015) suggest that large-scale gravitational destabilisation of the mush is one means to rapidly aggregate and release magmas to shallow levels. Conditions under which mushes become gravitational unstable, and the timescales of consequent magma ascent, are rich areas for further study.

## Electronic supplementary material

Below is the link to the electronic supplementary material.

**Supplementary Fig. A1** Plagioclase-melt Kd_Ca–Na_ ([Ca/Na]_plag_/[Ca/Na]_melt_) plotted as an exponential function of H_2_O^melt^ for basaltic andesite experiments at 2.4 (red) and 4 kbar (black) described in the text. Error bars are 1 s.d. The equation of the weighted best-fit line is given. The grey ovals are the corresponding Kd_Ca–Na_ values from Sisson and Grove (Sisson and Grove 1993a) for basaltic compositions. The slope defined by these data is the same as the fitted line, but displaced to higher values, demonstrating the compositional sensitivity of Kd_Ca–Na_. Consequently, the equation provided here should only be used for basaltic andesite compositions similar to the starting materials in Table 2. This equation was used in the construction of Fig. 13d. (EPS 320 kb)

**Supplementary Fig. A2** Compilation of experimental data showing An content of plagioclase stability as a function of H_2_O^melt^, temperature and pressure. The data set as on Fig. 14 (EPS 461 kb)

Supplementary material 3 (XLSX 28 kb)

Supplementary material 4 (XLSX 59 kb)

Supplementary material 5 (XLSX 40 kb)

Supplementary material 6 (XLSX 33 kb)

Supplementary material 7 (XLSX 33 kb)
